# Induced Manipulation of Atomically Dispersed Cobalt through S Vacancy for Photocatalytic Water Splitting: Asymmetric Coordination and Dynamic Evolution

**DOI:** 10.1002/advs.202405137

**Published:** 2024-08-13

**Authors:** Meixue Chen, Minhao Li, Shuqu Zhang, Xia Liu, Lixia Yang, Ren‐Jie Song, Jian‐Ping Zou, Shenglian Luo

**Affiliations:** ^1^ Key Laboratory of Jiangxi Province for Persistent Pollutants Prevention Control and Resource Reuse Nanchang Hangkong University Nanchang Jiangxi Province 330063 P. R. China; ^2^ College of Chemistry and Chemical Engineering Qingdao University Qingdao Shandong Province 266071 P. R. China

**Keywords:** atomic‐level Co, asymmetric coordination, S vacancy, regional separation of reaction sites, photocatalytic water splitting

## Abstract

It is still a challenge to construct single‐atom level reduction and oxidation sites in single‐component photocatalyst by manipulating coordination configuration for photocatalytic water splitting. Herein, the atomically dispersed asymmetric configuration of six‐coordinated Co‐S_2_O_4_ (two exposed S atoms, two OH groups, and two Co─O─Zn bonds) suspending on ZnIn_2_S_4_ nanosheets verified by combining experimental analysis with theoretical calculation, is applied into photocatalytic water splitting. The Co‐S_2_O_4_ site immobilized by Vs acts as oxidation sites to guide electrons transferring to neighboring independent S atom, achieving efficient separation of reduction and oxidation sites. It is worth mentioning that stabilized Co‐S_2_O_4_ configuration show dynamic structure evolution to highly active Co‐S_1_O_4_ configuration (one exposed S atom, one OH group, and three Co─O─Zn bonds) in reaction, which lowers energy barrier of transition state for H_2_O activization. Ultimately, the optimized photocatalyst exhibits excellent photocatalytic activity for water splitting (H_2_: 80.13 µmol g^−1^ h^−1^, O_2_: 37.81 µmol g^−1^ h^−1^) and outstanding stability than that of multicomponent photocatalysts due to dynamic and reversible evolution between stable Co‐S_2_O_4_ configuration and active Co‐S_1_O_4_ configuration. This work demonstrates new cognitions on immobilized strategy through vacancy inducing, manipulating coordination configuration, and dynamic evolution mechanism of single‐atom level catalytic site in photocatalytic water splitting.

## Graphical abstract

The atomically dispersed Co immobilized by S vacancy (Vs) inducing on ZnIn_2_S_4_ nanosheets reveal a unique asymmetric configuration of suspending six‐coordinated Co‐S_2_O_4_, which acts as oxidation sites to achieve efficient separation of reaction and oxidation sites and show the dynamic and reversible structure evolution to highly active Co‐S_1_O_4_ configuration, lowering the energy barrier of transition state for H_2_O activization.

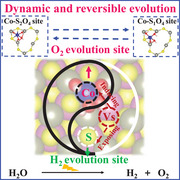

## 1 Introduction

With the increasing global energy demands and environmental issues, the development of renewable and ecofriendly energy sources has become extremely indispensable.^[^ ^1^, ^2^ ^]^ The utilization and conversion of inexhaustible solar energy to hydrogen energy from water has been investigated intensively.^[^ ^3^, ^4^, ^5^ ^]^ Up to now, various organic/inorganic semiconductors have been developed as efficient photocatalyst systems for hydrogen production. A major reason for the unsatisfactory H_2_ production efficiency is the inefficient separation and migration of photoexcited carriers.^[^ ^6^, ^7^, ^8^ ^]^ Therefore, the development of photocatalyst systems have focused on preventing the recombination of photogenerated charge carriers to improve photocatalytic performance of hydrogen evolution reaction (HER)/water splitting.

In recent years, metal sulfide photocatalysts,^[^ ^9^, ^10^ ^]^ especially multinary metal sulfide ZnIn_2_S_4_, have attracted great research attention in HER applications due to tunable band structure, excellent activity, and favorable photostability.^[^ ^11^, ^12^ ^]^ Researchers have made great efforts to optimize the photocatalytic activity of ZnIn_2_S_4_‐based photocatalysts. Doping engineering inject new vitality to the photocatalytic reaction by regulating available charge flow at the atomic level, achieving excellent and stable photocatalytic performance.^[^ ^13^ ^]^ Xie et al. reported the increased average lifetime of photoexcited electrons in oxygen doped ZnIn_2_S_4_ nanosheets for efficient photocatalytic HER.^[^ ^14^ ^]^ It is also reported that foreign metal doping can not only accelerate charge separation, but also favor adsorption and activation of H_2_O molecules because of the metal atoms enriched with positive charges. Pan et al. reported that Ag atom implanted into ZnIn_2_S_4_ nanosheet shows superior photocatalytic activity for water splitting compared to Cu atom due to the better adsorption energy for H_2_O molecules (Ag: −2.07 and Cu: −0.32 eV, respectively).^[^ ^15^ ^]^ It is clearly that the photocatalytic activity for water splitting is extremely limited by the species of dopant atoms for lattice/implanted doping in doping engineering. In essence, there is no doubt that the activity of catalytic sites is affected immensely by the coordination configuration.

Photogenerated carrier migration can also be efficiently regulated by the defects/vacancies engineering of semiconductor photocatalysts besides of doping engineering.^[^ ^16^ ^]^ Vacancies, regarded as a type of extremely delicate defect, are acknowledged to be skillfully deflected in general catalytic modulation. Jia et al. reported that Sn‐vacancy and S‐vacancy co‐modulate to form interfacial mediation, which can separate the photogenerated charge‐carriers and achieve high‐efficiency photocatalytic hydrogen evolution.^[^ ^17^ ^]^ The independent doping strategy or vacancy construction are two effective paths to enhance the HER performance of ZnIn_2_S_4_ photocatalyst. There are reasons to believe that the combination of these two strategies can play a synergistic effect for further improving HER performance. Zhang et al. reported a self‐adapting S vacancy (Vs) induced with atomic Cu implanted into lattice show a synergistic effect for adjusting regional separation of photogenerated charge due to the Cu dopant being a hole trap and Vs being an electron trap.^[^ ^18^ ^]^ Therefore, constructing vacancy/defects to expose the inner metal atoms is an important strategy for modulating carrier separation. It is well known that single‐atom catalysts (SACs) can expose catalytical metal active sites furthest, achieving metal utilization as high as 100% theoretically. However, the catalytic activity and stability are greatly affected by their coordination environment, as the high surface energy of single atoms is highly mobile and prone to form aggregates during synthesis and catalysis.^[^ ^19^, ^20^, ^21^, ^22^ ^]^ Likewise, defect metastability can considerably decrease the photocatalyst stability.^[^ ^23^, ^24^ ^]^ Interestingly, the wise structures obtained by placing single atoms (SAs) on vacancy site accurately can resolve the stability problems of SAs and metastability of vacancy due to coordination unsaturation and localized electron delocalization for vacancy/defect region.^[^ ^25^, ^26^ ^]^ Therefore, it is necessary to construct doped single atomic photocatalyst systems by grafting strategy through external vacancy inducing to resolve instability problems of the single atom/vacancy and activate water molecules for efficient photocatalytic water splitting.

In this study, we develop a rational structure of atomically dispersed Co immobilized through S vacancy inducing on ZnIn_2_S_4_ nanosheets, which is applied into photocatalytic water splitting. The ZnIn_2_S_4_ nanosheets with gradient Vs contents could be prepared controllably by adjusting solvent ratio in the solvothermal process. The structural configuration and coordination environment of Co atom are confirmed systematically from experimental results and theoretical calculations. The identified Co atoms and adjacent S atoms act as the reaction sites for oxidation and reduction respectively to achieve site separation. The optimized photocatalyst exhibits excellent photocatalytic activity for hydrogen evolution (H_2_: 11.36 mmol g^−1^ h^−1^) and water splitting (H_2_: 80.13 µmol g^−1^ h^−1^, O_2_: 37.81 µmol g^−1^ h^−1^) and outstanding stability. Finally, the mechanism of photocatalytic water splitting is proposed by analyzing energy barrier of water adsorption, activization, and evolution from theoretical calculations based on dynamic evolution of asymmetric Coordination for active sites.

## 2 Result and Discussion

The Vs‐ZIS@Co‐3 is synthesized by a simple two‐step hydrothermal method. Firstly, the Vs‐ZIS with gradient S vacancies was prepared in ethanol/water mixed solvothermal method. Secondly, ZnIn_2_S_4_ with S vacancies was soaked in Co^2+^ solution, Co SAs conjugated with OH groups were successfully immobilized on Vs‐ZIS nanosheets eventually. The macromorphology of ZIS, Vs‐ZIS and Vs‐ZIS@Co‐3 were characterized by scanning electron microscopy (SEM) (Figure S1, Supporting Information). It is apparent that all photocatalysts appear flower‐like 3D structure, which is hierarchically assembled by many cross‐linked nanosheets. Specific surface areas were conducted by nitrogen adsorption–desorption isotherms to further investigate morphology features (Figure S2, Supporting Information). The specific surface area of Vs‐ZIS@Co‐3 is 51.825 m^2^ g^−1^, which is the biggest in all samples (ZIS: 36.868 m^2^ g^−1^ and Vs‐ZIS: 44.752 m^2^ g^−1^). The increased specific surface areas mean more active sites exposure. X‐ray diffraction (XRD) patterns were performed to get the microscopic morphology of ZIS, Vs‐ZIS, and Vs‐ZIS@Co‐3 (Figure S3, Supporting Information). All catalysts are indexed to hexagonal ZnIn_2_S_4_ phase (JCPDS No. 65‐2023) and their characteristic peaks at around 21.59°, 27,69°, 30.45°, 39.77°, 47.18°, 52.44°, 55.58°, and 75.82°, corresponding to the {006}, {102}, {104}, {108}, {102}, {110}, {116}, {022}, and {212} lattice phases, respectively. No impurity diffraction peaks could be detected. It indicates that the addition of ethanol and Co do not change the crystal structure of ZIS. The microstructure was further investigated by TEM and HRTEM. The flower‐like hierarchical structure assembled by cross‐linked nanosheets is further confirmed (Figure S4, Supporting Information). Significant lattice interruptions and atomic defects labeled can be observed in Vs‐ZIS (Figure S5, Supporting Information), suggesting wide Vs distribution. The lattice spacing of Vs‐ZIS@Co‐3 is the same with ZIS (0.32 nm, {102}) (Figure S6, Supporting Information). The atomic ratio of Zn, In, and S for ZIS can be calculated as 1:1.74:3.65, which is close to the theoretical value from X‐ray spectroscopy elemental mappings (EDS) from TEM (Figure S7, Supporting Information). In comparison, the atoms ratio in Vs‐ZIS is 1:1.10:2.10, which suggest S atom absence (Figure S8, Supporting Information).

**Figure 1:**
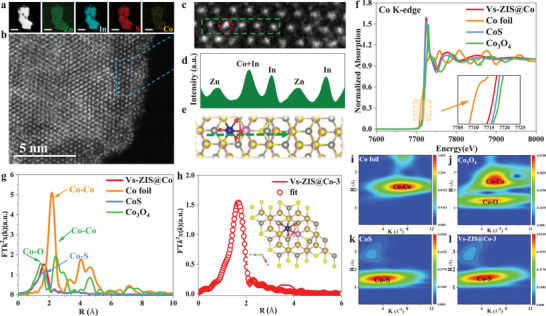
Morphology and fine‐structure characterizations of the Vs‐ZIS@Co‐3. a) Elemental mapping images. (Scale bar: 1 µm). b) Representative magnified HAADF‐STEM image. c) Enlarged HAADF‐STEM images obtained from the blue rectangle in (b). d) Line‐scanning intensity profiles from (c). e) The simulation structure (Zn (gray), In (purple), S (yellow), Co (blue), and O (red)). f) XANES spectra at Co K‐edge (The inset image is the magnified pre‐edge XANES region). g) Fourier‐transformed EXAFS spectra. h) Fitting curve of EXAFS spectra (The inset image is the optimized atomic model with Co‐S_1_O_4_ configuration). Wavelet transform of i) Co foil, j) Co_3_O_4_, k) CoS, and l) Vs‐ZIS@Co‐3.

The elemental mapping spectra from TEM reveal the highly uniform distribution of Co, Zn, In, and S elements in Vs‐ZIS@Co‐3 (**Figure** **1**a), which indicates the homogeneous dispersion of Co species without any discernible metallic agglomeration. It is necessary to determine the spatial position and coordination environment for the Co atom introduced in Vs‐ZIS. From visualizing perspective, aberration‐corrected high‐angle annular dark‐field scanning TEM (HAADF‐STEM) was conducted. The presence of isolated Co atoms on Vs‐ZIS support is clearly shown, marked by blue rectangles (Figure 1b). The specific atom shows the enhanced luminance in all the selected atoms, which is due to space superposition of In atom and introduced Co atom after comprehensive analysis of intensity profile and the simulation structure (Figure 1c–e). Subsequently, the valence and local coordination environments of Co in Vs‐ZIS @Co‐3 were investigated by Co k‐edge X‐ray absorption spectroscopy (XAS) (Figure 1f). The K‐edge absorption position of Vs‐ZIS@Co‐3 locates between that of Co (+0) and CoS (+2). The difference in the position and signal intensity (≈7710 eV) indicates that Co atom in Vs‐ZIS@Co‐3 have a different coordinated environment compared to all the standard samples. Fourier transforms (FT) of the extended X‐ray adsorption fine structure (EXAFS) oscillations (Figure 1g) shows that Vs‐ZIS@Co‐3 exhibits a dominant peak at 1.67 Å, which indicates the coexistence of Co─O bond (≈1.55 Å) bond and Co─S bond (≈1.85 Å) for Co coordination configuration.^[^ ^27^ ^]^ The absence peak of the Co─Co bond in Vs‐ZIS@Co‐3 materials compared with all the standard samples, verifying coordination configuration at the single atomic level. Besides, the coordinated peak position of Co located between Co─O and Co─S bond in Vs‐ZIS@Co‐3 means Co atom co‐coordinated with O atom and S atom jointly.^[^ ^28^ ^]^ Co atom bonded with two S atoms and four O atoms to be asymmetric configuration of six‐coordinated Co‐S_2_O_4_ are confirmed in after summarizing the quantitative structural fitting parameters of Co foil, Co_3_O_4_, CoS, and Vs‐ZIS @Co‐3 (Table S1, Supporting Information). The corresponding EXAFS fitting curves displayed in Figure 1h. Wavelet transforms (WT) of Co K‐edge EXAFS oscillations were performed to provide accurate resolution in k and R space (Figure 1i and S9, Supporting Information) to gain visual illustrations of Co coordination environment. The WT counter curve of Vs‐ZIS@Co‐3 presented a maximum signal at 4.5 Å^−1^, which was located differently with the Co foil (7.1 Å^−1^), Co_3_O_4_ (6.2 Å^−1^), and CoS (3.7 Å^−1^), demonstrating coexisting appearance of the Co─S bond and Co─O bond in Vs‐ZIS@Co‐3. What is more, it strongly suggests that Co atom do not substitute lattice atom (Zn or In) of ZIS.^[^ ^29^ ^]^ Element content analysis further verifies Co atom introduction does not be implanted into ZIS substituting any lattice atoms due to nearly invariable content of Zn and In atom (Table S2, Supporting Information). Finally, Vs‐ZIS photocatalyst with asymmetric configuration of six‐coordinated Co‐S_2_O_4_ of single atom level, different from introducing strategy of lattice substitution, was prepared and demonstrated successfully.

**Figure 2:**
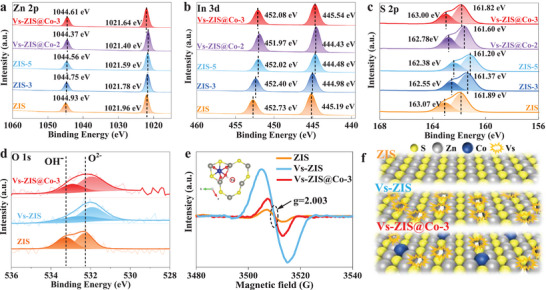
High‐resolution XPS spectra of a) Zn 2p, b) In 3d, c) S 2p, and d) O 1s for several photocatalysts. e) EPR spectra and f) the diagram of surface atomic arrangement of ZIS, Vs‐ZIS, and Vs‐ZIS@Co‐3 (The inset image is the optimized atomic model of filled Vs region by Co‐S_2_O_4_).

The elemental composition and electronic states of all samples were analyzed by X‐ray photoelectron spectroscopy (XPS) measurements (Figure S10, Supporting Information). For ZIS, the peaks at 1021.96 and 1044.93 eV can be corresponded to Zn 2p_3/2_ and 2p_1/2_ for Zn^2+^, respectively, the peaks at 445.19 and 452.73 eV can be indexed to the In 3d_5/2_ and 3d_3/2_ for In^3+^, respectively, and the peaks located at 161.89 and 163.07 eV can be assigned to S 2p_3/2_ and 2p_1/2_ for S^2−^, respectively (**Figure** **2**a–c). Compared with ZIS, the binding energies in Vs‐ZIS all show a slight shift toward lower binding energy for Zn 2p, In 3d and S 2p, which can be contributed to low‐coordination Vs existence.^[^ ^30^ ^]^ On the contrary, the binding energy in Vs‐ZIS@Co shift to higher binding energy for S 2p with increasing Co introduction. Simultaneously, the binding energy of Zn 2p and In 3d in Vs‐ZIS@Co‐3 is shifted towards higher energy levels compared to Vs‐ZIS. It can be concluded that the six‐coordinated Co‐S_2_O_4_ configuration can interact with ZIS framework powerful as the same as S atoms. There are reasons to believe that Co atom have been stabilized extremely, which is expected to present excellent stable performance. The O 1s spectra peaks for ZIS samples are shown at around 532.21 and 533.22 eV, attributing to O^2−^ and OH^−^, respectively (Figure 2d). It can be clearly shown that O 1s of Vs‐ZIS and Vs‐ZIS@Co‐3 are all shifted to lower binding energy compared to ZIS. Whereas, Vs‐ZIS@Co‐3 shift toward higher binding energies compared with Vs‐ZIS, which is attributed to the strong interaction of Co with O on the surface of Vs‐ZIS.^[^ ^31^ ^]^ The Co 2p presents two shake‐up peaks (Co*^x^* ^+^) at 781.21 and 796.18 eV (Figure S11, Supporting Information).^[^ ^32^ ^]^ It further validates that Co single atom with positive oxidation state has a strong interaction with the Vs‐ZIS support. In contrast, the characteristic peak of Co metal 2p_3/2_ for Vs‐ZIS@Co‐4 is observed, indicating the existence of metallic state.^[^ ^33^ ^]^ The Co atomic contents in Vs‐ZIS@Co‐*y* (*y* = 1–4) sample were quantified to be 0.34%, 0.72%, 0.99%, and 1.57%, respectively, from XPS analysis (Table S2, Supporting Information).

The electron paramagnetic resonance (EPR) spectrum and Raman vibrational modes were conducted to further investigate the impact on electronic states for the surface atomic arrangement and property. The intense signal at *g* ≈ 2.003 further shows the presence of Vs in Vs‐ZIS and Vs‐ZIS@Co‐3 (Figure 2e). Vs concentration can be confirmed by comparing the S atom and In atom for normal levels of In atoms in all the samples. The S/In atomic ratio is calculated to be 2.04, 2.00, 1.93, 1.93, 1.96, 2.00, and 2.01 for ZIS, ZIS‐3, ZIS‐5, and Vs‐ZIS@Co‐y samples. The Vs concentration can be calculated to be 0, 1.96%, 5.39%, 5.39%, 3.92%, 1.96%, and 1.47% for corresponding samples, respectively (Table S3, Supporting Information). The Vs‐ZIS@Co‐3 photocatalyst showed the reduced concentration of S vacancies after Co introduction, which strongly indicated Co sewing and filling S vacancies under its inducing role. The calculated partial charge densities show that the positive charge density of Zn atoms around the Vs region are significantly higher than that of the other Zn atoms (Figure S12, Supporting Information). Therefore, Co(OH)*_x_^y^* ^−^ is induced to adsorb on the Vs region by electrostatic attraction. The exposed Zn due to Vs formation is easier to bond with OH in Co(OH)*_x_^y^* ^−^ to form Zn─O, which cause the Zn 2p shift. The pure ZIS of Raman vibrational modes are found at 125, 248, 305, 344, and 365 cm^−1^ (Figure S13, Supporting Information). The strong band at 125 cm^−1^ demonstrates the layered structure of hexagonal ZIS due to low‐frequency rigid‐layer characteristic of anisotropic nanosheets. The other four peaks can be sequentially ascribed to longitudinal optical mode (LO_1_), transverse optical mode (TO_2_), longitudinal optical mode (LO_2_), and A_1g_ mode for ZIS nanosheet respectively.^[^ ^13^ ^]^ No other crystal impurities peaks were detected. These four characteristic peaks of Vs‐ZIS show broadened and depressed intensity due to Vs existence resulting in atomic disordered arrangement. The LO_1_ mode for Vs‐ZIS@Co‐3 presents slight enhancement compared with Vs‐ZIS, indicating the improved crystallinity degree, which can be attributed to the Co‐S_2_O_4_ configuration compensating Vs region. It is worth noting that ZIS@Co‐3 (without surface defects) and Vs‐ZIS@Co‐3 hold the same characteristic peaks. Co single atom is not completely embedded in the lattice plane of Vs‐ZIS (Figure 2f). It is definite that Co‐S_2_O_4_ configuration suspends on Vs‐ZIS surface, which establish interaction by O and S atoms under Vs inducing role based on above systematic and comprehensive analysis. The proposed and constructed atomic structure of Co in Vs‐ZIS@Co‐3 (Figure 1e,h) is reasonable and ideal through theoretical simulation.

It is necessary to analyze bandgap structure to discuss the charge potential energy. The optical properties of all catalysts observed from UV–vis diffuse reflectance spectra (Figure S14a, Supporting Information). ZIS sample presents an absorption edge located at about 550 nm, and the other photocatalysts show a significant redshift with the introduction of vacancies/Co. The bandgap of ZIS, ZIS‐3, ZIS‐5, Vs‐ZIS@Co‐2, and Vs‐ZIS@Co‐3 are calculated to be 2.77, 2.73, 2.66, 2.63, and 2.52 eV, respectively, by using the Kubelka–Munk function (Figure S14b, Supporting Information). Vs‐ZIS presents a slightly narrower bandgap compared with ZIS due to the introducing Vs.^[^ ^34^ ^]^ The bandgap of Vs‐ZIS nanosheets narrows gradually with Co introduction. The valence band (VB) position could be estimated by XPS analysis (Figure S14c, Supporting Information), and the conduction band (CB) position further could be calculated. Finally, bandgap structures of all samples were displayed, which all are capable of water splitting (Figure S14d, Supporting Information). It is important to further to understand charge separation behaviors deeply in photocatalytic hydrogen evolution/water splitting. The steady‐state photoluminescence (PL) spectra demonstrated that Vs‐ZIS@Co‐3 has the lowest intensity, verifying satisfied charge separation (Figure S15a, Supporting Information). The average PL lifetime (*τ*_ave_) differences of all samples could be obtained according to the calculated equation (Table S4, Supporting Information). It shows that the PL lifetime of Vs‐ZIS@Co‐3 (*τ*_ave_ = 1.43 ns) is the longest among all samples (Figure S15b, Supporting Information). Similarly, the enhanced photocurrent intensity indicates the better performance of carrier separation in Vs‐ZIS@Co‐3 (Figure S15c, Supporting Information). The Vs‐ZIS@Co‐3 exhibits fast interfacial charge transfer property from the smallest radius in electrochemical impedance spectroscopy (Figure S15d, Supporting Information). All in all, the constructed Vs‐ZIS@Co‐3 structure with atomic interface of Co‐S_2_O_4_ configuration could separate photogenerated charges efficiently.

**Figure 3:**
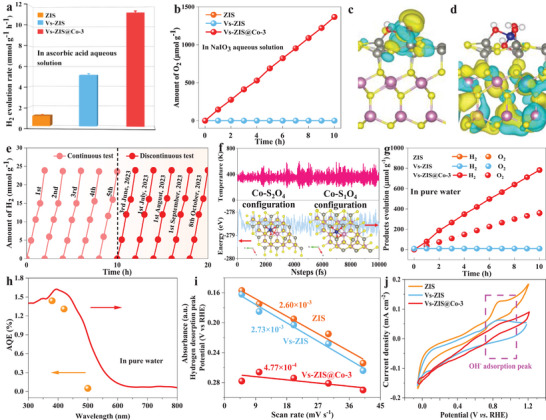
Performance comparison of ZnIn_2_S_4_, Vs‐ZIS, and Vs‐ZIS@Co‐3 for a) H_2_ evolution half‐reaction and b) O_2_ evolution half‐reaction (Sacrificial agent: 0.5 m ascorbic acid aqueous solution in HER and 0.02 m NaIO_3_ aqueous solution in OER, 300 W xenon lamp, λ > 420 nm). Charge density distribution of c) VBM and d) CBM of Vs‐ZIS@Co‐3 with Co‐S_2_O_4_ configuration (side view). The yellow and blue regions represent the accumulation and consumption of electrons, respectively. e) Photocatalytic recycling test of hydrogen evolution and f) the evolution of total electronic energy and temperature during time‐step simulation of Vs‐ZIS@Co‐3. The time‐step in Molecular Dynamics (MD) simulations is 1 fs (The inset images are the optimized Co‐S_2_O_4_ and Co‐S_1_O_4_ configuration). g) Photocatalytic water splitting performance with the increasing irradiation time (300 W xenon lamp, λ > 420 nm). h) Wavelength‐dependent AQE in pure water for Vs‐ZIS@Co‐3. i) Plots of hydrogen desorption peak position versus scan rates, and j) cyclic voltammetry of ZIS, Vs‐ZIS, and Vs‐ZIS@Co‐3.

The photocatalytic performance of the designed photocatalysts was firstly proceeded with ascorbic acid as a sacrificial reagent under visible‐light irradiation (*λ* > 420 nm). As observed in Figure S16 (Supporting Information), Vs‐ZIS samples show the better photocatalytic performance for hydrogen evolution compared with ZIS (1.02 mmol g^−1^ h^−1^) due to suitable Vs introduction accelerating charge separation. The H_2_ evolution rate reaches to the best value (5.13 mmol g^−1^ h^−1^) when Vs concentration is adjusted at 5.39% (Table S3, Supporting Information). HER performance decreases with Vs concentration further increasing. More Vs introduction play the roles of electrons traps to promote carriers separation in ZIS no more, become recombination centers. The photocatalytic performance of all Vs‐ZIS@Co‐*y* photocatalysts is enhanced significantly with atomic Co‐S_2_O_4_ configuration fixation (Figure S17, Supporting Information). In detail, Vs‐ZIS@Co‐3 achieved the best H_2_ evolution rate of 11.36 mmol g^−1^ h^−1^. What is mentioned, excessive Co introduction reduces photocatalytic activity for H_2_ evolution as shown from Vs‐ZIS@Co‐4. This may be the fact that excess Co inevitably and partially deposited on the intact surface without Vs, leading to Co agglomeration and reduced photocatalytic activity, which is also verified from the metallic Co state in Vs‐ZIS@Co‐4 (Figure S11, Supporting Information). The examined photocatalytic activity of Vs‐ZIS@Co‐3 (11.36 mmol g^−1^ h^−1^) and ZIS@Co‐3 (no surface Vs) (3.26 mmol g^−1^ h^−1^) with same Co contents showed that appropriate concentration of Co and Vs play the synergetic role for the photocatalytic performance (Figure S18, Supporting Information). Finally, the H_2_ evolution rate for Vs‐ZIS@Co‐3 is approximately two and five times than that of Vs‐ZIS and ZIS, respectively (**Figure** **3**a). In order to better understand the process of water splitting by photocatalysts under pure water conditions, H_2_O was substituted by D_2_O to verify proton source by isotopic labeling, and the product traces were identified by gas chromatography‐mass spectrometry (GC‐MS) (Figure S19, Supporting Information). It can be clearly seen that there are fragments with large m/z values on Vs‐ZIS@Co‐3 under D_2_O conditions. There is no doubt that protons from H_2_O are involved in the photocatalytic water splitting. The real Co loading contents of Vs‐ZIS@Co‐3 were determined from inductively coupled plasma mass spectrometry (ICP‐MS) and found to be 0.66 wt% (Figure S20, Supporting Information). Visible‐light‐driven OER performance was proceeded in NaIO_3_ aqueous solution, which acts as electron sacrificial reagents (Figure 3b). The O_2_ releasing rate is up to 136.7 µmol g^−1^ h^−1^ for Vs‐ZIS@Co‐3 photocatalyst. By contrast, no O_2_ gas is detected for Vs‐ZIS and ZIS photocatalysts in the same reaction conditions. Charge density distributions of VB maximum (VBM) and CB minimum (CBM) for Vs‐ZIS@Co‐3 were conducted to investigate the charges separation situation after Co‐S_2_O_4_ configuration introduction (Figure 3c,d). It can be found that holes are mainly consumed at Co and Zn atoms in the VBM, while electrons are mainly accumulated by Vs and In atoms in the CBM. It can be inferred that the oxidation reaction probably occurs in Co region, while the reduction reaction mainly occurs in Vs region. The reaction sites for oxidation and reduction are separated regionally after the suspended six‐coordinated Co‐S_2_O_4_ configuration introduction. Single Co atoms acts as an electron pump and Vs acts as an electron trap, which synergistically promotes charge separation and transfer efficiency. Besides, Vs‐ZIS@Co‐3 photocatalyst maintains excellent stability in both continuous testing and discontinuous testing (Figure 3e). No obvious activity degradation for HER was observed in 10 h measurement and long‐dormant sample. The thermodynamic stability of Vs‐ZIS@Co‐3 was also evaluated from Molecular Dynamics (MD) simulation (Figure 3f). The MD results show that Vs‐ZIS@Co‐3 is stable at 360 k and its maximum energy change is only 1 eV, which strongly verifies the stability of the structure. It is worth mentioning that stabilized Co‐S_2_O_4_ site (Co bonded with two exposed S atoms, two independent OH groups, and two Co─O─Zn bonds) show the dynamic structure evolution to highly active Co‐S_1_O_4_ site (Co bonded with one exposed S atom, one independent OH group, and three Co─O─Zn bonds) in reaction. In experiment, the leaching rate of metal ions in solution was detected by ICP‐MS after the photocatalytic test. The corresponding data of metal ion leaching efficiency (Table S5, Supporting Information) were obtained through the calibration curve (Figure S20, Supporting Information). Vs‐ZIS@Co‐3 photocatalysts exhibit the lowest metal ion leaching efficiency after four photocatalytic cycle tests. High‐resolution XPS spectrum of Co 2p for used Vs‐ZIS@Co‐3 still maintain the same with the fresh one (Figure S21, Supporting Information). All these analysis from theoretical simulation and experiment data indicates proposed and constructed atomic structure Co‐S_2_O_4_ site hold excellent photocatalytic activity and stability.

The photocatalytic performance for overall water splitting over Vs‐ZIS@Co‐3 was carried out without any sacrificial reagents or co‐catalysts with encouraged by the above discussion. Notably, the H_2_ and O_2_ evolution rate of Vs‐ZIS@Co‐3 is 80.13 and 37.81 µmol g^−1^ h^−1^, respectively, with H_2_:O_2_ ratios very close to the expected 2:1 stoichiometry (Figure 3g). No gas is detected in Vs‐ZIS and ZIS under the same conditions. The performance comparison was listed in this work and some representative photocatalyst systems from 2018 (Figure S22 and Table S6, Supporting Information). Specifically, Vs‐ZIS@Co‐3 exhibits superior photocatalytic performance with higher economic effect compared to some representative multicomponent composite photocatalysts. The photocatalytic performance of Vs‐ZIS@Co‐3 is also better compared to ZIS‐based photocatalysts, convincingly confirming the excellent activity of water splitting in this field. The excellent photocatalytic performance for water splitting over Vs‐ZIS@Co‐3 benefit for the overall separation of oxidation and reduction sites. The apparent quantum efficiency (AQE) over Vs‐ZIS@Co‐3 in pure water is 380, 420, and 500 nm was 1.44%, 1.31%, and 0.05%, respectively, which is listed in Table S7 (Supporting Information). The AQE calculation process was described in Text S1 (Supporting Information). The respective AQE at different monochromatic wavelengths show the same trend with the light absorption properties of Vs‐ZIS@Co‐3 (Figure 3h). Solar‐to‐hydrogen conversion efficiency (STH) was described in Text S2 (Supporting Information). It is necessary to investigate hydrophilicity and adsorption behavior of photocatalyst from macroscopic surface and microscopic sites. The hydrophilicity was examined via the static water contact angle (θ) measurements (Figure S23, Supporting Information). All photocatalysts exhibit a degree of hydrophilicity (θ < 90°). The better hydrophilicity for Vs‐ZIS@Co‐3 compared with ZIS@Co‐3 confirm that the single atomic Co are anchored on Vs‐ZIS by hydrophilic group of Co‐S_2_O_4_ certainly. The better hydrophilicity is conducive to higher specific surface and more exposed surface, which can benefit for photocatalytic water splitting. Cyclic voltammetry (CV) measurement at different scan rates was performed to analyze the kinetics of hydrogen adsorption/desorption behavior for catalysts (Figure S24, Supporting Information). The redox peak position of Vs‐ZIS@Co‐3 shift smaller significantly compared to ZIS and Vs‐ZIS, implying the faster hydrogen adsorption/desorption.^[^ ^35^ ^]^ Therefore, the hydrogen desorption kinetics could be quantified by plotting peaks position of hydrogen desorption versus scan rate and comparing the fitted slopes (Figure 3i). The fitted slope of Vs‐ZIS (2.73 × 10^−3^) is close to that of bare ZIS (2.60 × 10^−3^), indicating the unaltered hydrogen desorption kinetics. In contrast, the lower slope of Vs‐ZIS@Co‐3 (4.77 × 10^−4^) indicates that hydrogen desorption kinetic is accelerated substantially. Similarly, CVs were also used to study the adsorption behavior of OH^−^ on photocatalyst surfaces (Figure 3j). The characteristic peak of OH adsorption (OH_ad_) around 0.85 V_RHE_ was observed on Vs‐ZIS and ZIS. The larger peak area of Vs‐ZIS suggest that OH_ad_ is associated with the formation of M─OH bonds on photocatalysts.^[^ ^36^ ^]^ The peak intensity of Vs‐ZIS@Co‐3 is weaker and reduced than that of Vs‐ZIS and ZIS. Appropriate interaction between OH_ad_ and Vs‐ZIS@Co‐3 facilitate intermediate transformation (OH_ad_ → OH^−^) to favor OER process. The excellent photocatalytic water splitting of Vs‐ZIS@Co‐3 benefits for charge and sites separation, hydrophilicity, and accelerated H and OH intermediates transformation due to constructed six‐coordinated Co‐S_2_O_4_ configuration by the above analysis.

**Figure 4:**
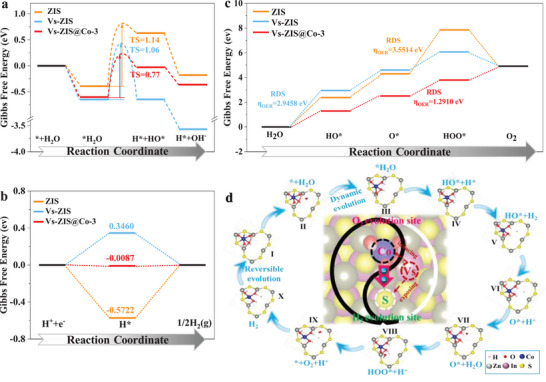
Free energy diagrams of the a) Volmer step, b) HER process, and c) OER process on ZIS, Vs‐ZIS, and Vs‐ZIS@Co‐3. d) Proposed photocatalytic mechanism for H_2_ and O_2_ evolutions from H_2_O based on dynamic and reversible evolution between Co‐S_2_O_4_ configuration and Co‐S_1_O_4_ configuration for Vs‐ZIS@Co‐3.

It is necessary to further elucidate mechanism of H_2_ and O_2_ evolution on the surface photocatalysts combined with theoretical calculation based on understanding of the structural properties and electronic behaviors. Table S8 (Supporting Information) listed optimized structural model in top view and side view of ZIS, Vs‐ZIS and Vs‐ZIS@Co‐3. In a typical Volmer step, which involves H_2_O adsorption and dissociation, processes the formation of adsorbed H (H*) and OH (HO*), H* and OH^−^. It is evident that the adsorption energy of Vs‐ZIS@Co‐3 and Vs‐ZIS for H_2_O are very close to each other (**Figure** **4**a). However, the energy barrier of transition state (TS) for H_2_O dissociation over Vs‐ZIS@Co‐3 (0.77 eV) is least compared with that of ZIS (1.14 eV) and Vs‐ZIS (1.06 eV), which means that Vs‐ZIS@Co‐3 hold the greatest capacity for photocatalytic water splitting process among three photocatalysts. The energy barrier for producing H* and OH^−^ over Vs‐ZIS@Co‐3 verge on zero indicates excellent adsorption–desorption kinetics of H* and OH^−^ during the reaction. Theoretical calculations were extended to elucidate the effect of Vs and Co atom on the interfacial HER and OER mechanism to gain a comprehensive insight into water splitting (Figure 4b,c). The optimized structural model of H* was listed for three photocatalysts in Table S9 (Supporting Information). The adsorption free energy of H* (Δ*G*_H*_) value of an ideal photocatalyst for HER should be tended to zero to balance adsorption and desorption kinetics from the point of bonding H* and H_2_ release on catalyst surface.^[^ ^37^ ^]^ Vs‐ZIS surface is more favorable for H_2_ release but not bonding H*, while ZIS is reverse from theoretical HER kinetics analysis (Figure 4b). ΔG_H*_ value of Vs‐ZIS@Co‐3 tends to zero, which reach to the best balance for H* adsorption and H_2_ desorption kinetics. The optimized optimum S site for bonding H* over Vs‐ZIS@Co‐3 is adjacent to Co‐S_2_O_4_ configuration around Vs region (Table S9, Supporting Information), which further impact with photo‐generated electrons localized by Vs region to perform reduction reaction. The constructed Co‐S_2_O_4_ configuration induced by Vs not only reduces the intrinsic adsorption–desorption barrier for HER, but also activates more active centers on basal plane to promote photocatalytic activity. The multisteps reaction from H_2_O adsorption to O_2_ generation reveals that intermediates containing O atoms (HO*, O*, and HOO*) are consistently bounded with positively charged metal atoms in all photocatalysts (Table S10, Supporting Information). It reveals that the rate‐determining step (RDS) of Vs‐ZIS@Co‐3 and Vs‐ZIS are identical for HOO* generation from O*, while ZIS is HO* generation from H_2_O (Figure 4c). Vs existence affects RDS greatly. The lowest energy barrier over Vs‐ZIS@Co‐3 (1.29 eV) compared to ZIS (2.38 eV) and Vs‐ZIS (2.95 eV) of generation O* from H_2_O suggests that the introduction of Co atoms weakened ability of OH^−^ adsorption, which is accordant with CVs results (Figure 3j). Obviously, the smaller η_OER_ (1.2910 eV) of Vs‐ZIS@Co‐3 implies the synergistic roles of unfilled Vs and Co‐S_2_O_4_ configuration in reducing energy barrier of OER process. The possible mechanism of photocatalytic water splitting was proposed over Vs‐ZIS@Co‐3 (Figure 4d). The first step involves the adsorption of H_2_O molecule onto the single atomic Co‐S_1_O_4_ configuration from structure evolution of Co‐S_2_O_4_ configuration by Vs inducing through breaking one Co─S bond. Subsequently, *H_2_O are formed and further split into HO* bonded with Co atom and H* bonded with adjacent S of Co‐S_1_O_4_ configuration, which is exposed under Vs existence. Electrons are transferring to the neighboring S directionally due to electron pump role of Co‐S_1_O_4_ configuration. This is followed by the rapid impacting conversion to H_2_ of two active H* groups for HER process. In the OER process, HO* is oxidized to O* by holes (*h* ^+^), O* then reacts with additional H_2_O to form HOO*, and finally HOO* is further oxidized to produce O_2_. It is worth noting that H^+^ is always generated during this process of HOO* → O_2_, which will combine with photogenerated electrons to form H* to perform HER process. This structural characteristics of Vs‐ZIS@Co‐3 promotes H_2_O oxidation while providing more H^+^ through H_2_O deprotonation to participate in HER process. Finally, the highly active Co‐S_1_O_4_ coordination configuration photocatalyst is restored to original stabilized Co‐S_2_O_4_ coordination configuration to prepare for the next cycle in the reaction. The process of photocatalytic water splitting over Vs‐ZIS@Co‐3 to generate H_2_ and O_2_ is given in the following equations

(1) $$*+H_{2}O\to H*+\mathrm{HO}*$$

(2) $$H*+H*\to H_{2}$$

(3) $$\mathrm{HO}*\to O*+H^{+}+e^{-}$$

(4) $$O*+H_{2}O\to \mathrm{HOO}*+H^{+}+e^{-}$$

(5) $$\mathrm{HOO}*\to *+O_{2}+H^{+}+e^{-}$$

## 3 Conclusion

In summary, single atomic Co confined on ZnIn_2_S_4_ nanosheets is successfully designed and applied into photocatalytic water splitting. Particularly, atomically dispersed Co is immobilized on the ZnIn_2_S_4_ nanosheets through S vacancies, resulting in asymmetric and six‐coordinated Co‐S_2_O_4_ configuration which suspended on Vs‐ZIS surface. The Vs‐ZIS@Co‐3 presents excellent stable performance due to powerful interaction between Co‐S_2_O_4_ configuration and Vs‐ZIS framework. The cyclic voltammetry measurements demonstrated that appropriate interaction between OH_ad_ and Vs‐ZIS@Co‐3 facilitate intermediate transformation (OH_ad_ → OH^−^) to favor OER process. Theoretical calculation indicates that stabilized Co‐S_2_O_4_ configuration show dynamic structure evolution to highly active Co‐S_1_O_4_ configuration (one exposed S atom, one OH group, and three Co─O─Zn bonds) in reaction. The unfilled Vs and Co‐S_1_O_4_ configuration play the synergistic roles in reducing energy barrier of transition state for HER and OER process. Ultimately, the optimized Vs‐ZIS@Co‐3 photocatalyst presents excellent activity of hydrogen evolution (11.36 mmol g^−1^ h^−1^), which is one order of magnitude of that for pure ZIS (1.02 mmol g^−1^ h^−1^), and shows excellent performance of photocatalytic water splitting (H_2_: 80.13 µmol g^−1^ h^−1^, O_2_: 37.81 µmol g^−1^ h^−1^) without any sacrificial agent, which is even much better than that of multicomponent photocatalyst systems. The excellent performance and outstanding ability of optimized Vs‐ZIS@Co‐3 are due to the dynamic and reversible evolution between stabilized Co‐S_2_O_4_ configuration and highly active Co‐S_1_O_4_ configuration.

## 4 Experimental Section

### Materials

Zinc acetate dihydrate (Zn(Ac)_2_·2H_2_O), indium (III) chloride tetrahydrate (InCl_3_·4H_2_O), thioacetamide (TAA), and cobalt chloride hexahydrate (CoCl_2_·6H_2_O) were obtained from Sinopharm Chemical Reagent Co., Ltd. (China). All chemical reagents were used without further purification in the experiments.

### Synthesis of ZnIn_2_S_4_ (ZIS) and ZnIn_2_S_4_ with Sulfur Vacancies (Vs‐ZIS)

To synthesize ZIS by a simple solvothermal method, 0.5 mmol of Zn(Ac)_2_·2H_2_O, 1 mmol of InCl_3_·4H_2_O, and 6 mmol of TAA were dissolved in 50 mL of deionized water solution with continuous stirring for 0.5 h. Then, the colorless mixture was maintained at 220 °C for 9 h in a 100 mL Teflon‐lined autoclave. The final yellow precipitate was obtained through washing by deionized water and ethanol for several times, centrifugation, and freeze‐dried for 12 h. Similarly, ZIS with different concentrations of S vacancies (ZIS‐*x*) was prepared by the same procedure with ZIS, except that all the raw materials were dissolved in a mixture solution (50 mL) with different ratios of deionized water and ethanol. For the ZIS‐*x* sample, when *x* = 1, 3, 5, 7, and 10, the volumes of ethanol added were 1, 3, 5, 7, and 10 mL, respectively. ZIS‐5 is abbreviated as Vs‐ZIS.

### Synthesis of Vs‐ZnIn_2_S_4_@Co‐*y* (Vs‐ZIS@Co‐*y*)

Vs‐ZIS@Co‐*y* were synthesized by an electrostatic adsorption method. 200 mg of Vs‐ZIS powder were added to 150 mL of deionized water and ultrasonic for 0.5 h. Then, different volumes of CoCl_2_·6H_2_O (4 mg mL^−1^) aqueous solution were added into above solution and stirred for 10 min. After that, the mixed solution was transferred to a three‐necked flask and heated and stirred at 70 °C for 8 h under a N_2_ atmosphere. After heating, the Vs‐ZIS@Co‐*y* composite powders were collected by centrifugation and washed three times using deionized water. The collected powders were freeze‐dried for 12 h and named as Vs‐ZIS@Co‐*y*. For the Vs‐ZIS@Co‐*y* sample, when *y* = 1, 2, 3, and 4, the volumes of CoCl_2_·6H_2_O added were 225, 450, 675, and 900 µL, respectively.

### Characterization

The morphological structures of all the photocatalyst samples were characterized by SEM. The detailed microstructures were observed at 200 kV using TEM (JEOL, JEM‐2100 F (HR)). HAADF‐STEM images were obtained from a FEI‐Titan Themis G2 60‐300 TEM/STEM with an aspherical aberration corrector. The surface properties were examined from contact‐angle measurement of a water droplet with a SDC‐350KS instrument. The BET adsorption and desorption isotherms were performed on a Belsorp‐Mini II analyzer (Japan). The XRD data of the as‐synthesized samples were obtained with a Bruker D8 Advance X‐ray diffractometer (Bruker, Germany) using Cu Kα radiation (λ = 1.5406 Å). The electronic structures and chemical states of elements measured by XPS measurements were acquired with a VG Escalab 250 spectrometer equipped with an Al anode (Al Kα = 1486.7 eV). The EXAFS measurements were carried out on the sample at beamline 07A1 of Taiwan Light Source (TLS) of National Synchrotron Radiation Research Center (NSRRC). This beamline adopted fixed‐exit double crystal Si (111) monochromator to ranging the X‐ray energy from 5 to 23 keV. The end‐station equipped three ionization chambers and Lytle detector for transmission and fluorescence mode X‐ray absorption spectroscopy. The beam size of X‐ray on the sample is about 0.5 × 0.25 mm (H × V) with flux higher than 1 × 10^10^ photon s^−1^. The EPR measurements were performed at room temperature using a spectrometer (Bruker, A300) at 300 K and 9.86 GHz. The DRS were acquired by a Cary 300 UV–vis spectrophotometer. The PL spectra were measured by a Hitachi F4500 fluorescence spectrophotometer at room temperature (excited wavelength at 340 nm). The TRPL spectra were recorded using a time‐resolved fluorescence spectrofluorometer (Edinburgh, FS5). Electrochemical measurements were performed on an electrochemical workstation (Chenhua Instrument Co., Shanghai) with a three‐electrode electrochemical system. An Ag/AgCl electrode, Pt electrode, and fluoride tin oxide (FTO) with covered as‐prepared samples (active area of 1.0 × 1.0 cm^2^) were used as the reference electrode, counter electrode, and working electrode, respectively. The transient photocurrent response was also evaluated under visible‐light irradiation (the interval is 50 s for light on and off) at a fixed potential of 0.24 V (vs Ag/AgCl) for 350 s. The electrochemical impedance spectroscopy (EIS) was conducted in 0.2 m Na_2_SO_4_ aqueous solution (pH = 6.8) using the above three‐electrode system. The CVs of adsorbed H were carried out in N_2_‐saturated 0.2 m Na_2_SO_4_ solution with the scan rate from 5 to 40 mV s^−1^, the electrodes used are the same as those described above. The CVs of adsorbed OH were carried out in N_2_‐saturated 7 m KOH solution with the scan rate maintained at 10 mV s^−1^. The prepared electrode was used as the working electrode, and the Hg/HgO electrode and the nickel mesh were used as the reference electrode and counter electrode, respectively. The preparation process of the working electrode: 2 mg of photocatalyst was ultrasonically dispersed in 50 µL of ethyl alcohol to obtain a suspension, which was then uniformly smeared onto the FTO glass substrate. Next, the coated FTO glass was dried at 60 °C. The concentrations of metal ions were detected from ICP‐MS (iCAP‐RQ, Thermo Fisher Scientific, USA). The isotope‐labeled experiments were performed using D_2_O instead of H_2_O, and the products were analyzed using gas chromatography–mass spectrometry (Agilent 7890A AMETEK DAC 200 MS).

### Evaluation of Photocatalytic Performance

Photocatalytic measurements were carried out using Labsolar‐III(AG) closed gas system (Perfectlight, Beijing, China) with a 100 mL quartz flask. Typically, in photocatalytic H_2_ evolution half‐reaction, 5 mg photocatalyst was dispersed into 100 mL of aqueous solution containing 0.2 m ascorbic acid as the sacrificial reagents for consuming photogenerated holes. Before reaction, the residual air in solution and pipeline was vacuumed for 30 min to remove air. 300 W Xenon‐lamp (PLS‐SXE300) equipped with 420 nm cut‐off optical filter was used as visible light source. The system temperature was controlled at 10 °C to eliminate the influence of temperature on photocatalytic reaction. The evolved gas was detected by a gas chromatography with Ar carrier gas (FLGC 9790II, TCD). Photocatalytic O_2_ evolution half‐reactions were carried out under the same conditions except that 0.02 m NaIO_3_ aqueous solution of 100 mL was adopted as the electron sacrificial reagent. Photocatalytic overall water splitting reactions were carried out under the same conditions except that the 30 mg photocatalyst powder was dispersed in 100 mL of distilled water without any sacrificial reagents.

## Conflict of Interest

The authors declare no conflict of interest.

## Supporting information

Supporting Information

## Data Availability

The data that support the findings of this study are available in the supplementary material of this article.
