# Supplementary material for: Induced Manipulation of Atomically Dispersed Cobalt through S Vacancy for Photocatalytic Water Splitting: Asymmetric Coordination and Dynamic Evolution
Source: Adv Sci (Weinh). 2024 Aug 13;11(39):2405137. doi: 10.1002/advs.202405137 (PMC11496988; doi:10.1002/advs.202405137)
Supplement: Supplementary file 1 — Supporting Information [file ADVS-11-2405137-s001.docx]

**Supporting Information**

Induced Manipulation of Atomically Dispersed Cobalt through S Vacancy for Photocatalytic Water Splitting: Asymmetric Coordination and Dynamic Evolution

Meixue Chen,^a^ Minhao Li,^a^ Shuqu Zhang,^a,^* Xia Liu,^b^ Lixia Yang,^a^ Ren-Jie Song,^a^ Jian-Ping Zou,^a^ Shenglian Luo^a^

*^a^* Key Laboratory of Jiangxi Province for Persistent Pollutants Prevention Control and Resource Reuse, Nanchang Hangkong University, Nanchang 330063, Jiangxi Province, People’s Republic of China

*^b^* College of Chemistry and Chemical Engineering, Qingdao University, Qingdao 266071, Shandong Province, People’s Republic of China

***Corresponding author:** Email: zhangshuqu2013@126.com (Shuqu Zhang)

**Calculation Methods**

All calculations were performed by using the VASP package. The Perdew-Burke-Ernzerhof (PBE) within the generalized gradient approximation (GGA) treats the exchange-correlation functional, while the projected augmented wave (PAW) describes the electron-ion interaction. Grimme’s zero-damping DFT-D_3_ method was used to calculate the van der Waals correction. For the plane-wave basis set, a cutoﬀ of E_cut_ = 500 eV has been used. For bulk ZnIn_2_S_4_, A Monkhorst–Pack *k*-point sampling of 5 × 5 × 1was selected. Meanwhile, the convergence criterion of the energy and force within the process of geometric optimization is chosen as 10^−5^ eV and 0.01 eV/Å, respectively. To eliminate the interaction, the vacuum region of 15Å was applied. The molecular dynamics (AIMD) simulation was carried using the Nosé-Hoover method is used for temperature control and the PBE/PAW method for electronic energy calculation. The time-step in the AIMD simulations was 1 fs. The adsorption free energy of H (ΔG_H_^0^) is evaluated the activity of photocatalytic hydrogen production. And ΔG_H_^0^ is used to be roughly calculated as: ΔG_H_^0^ = ΔE_H_ + 0.28eV, where ΔE_H_ is the differential binding energy is often used to describe the stability of H atoms, ΔE_H_ = E(ZnIn_2_S_4_ + H) − E(ZnIn_2_S_4_) − 1/2E(H_2_), where E(ZnIn_2_S_4_+ H) is the total energy for ZnIn_2_S_4_ with one adsorbed H atom, E(ZnIn_2_S_4_) represents the total energy for the photocatalyst without H atoms, and E(H_2_) is the energy of a gas phase H_2_. Transition states were searched by nudged elastic-band method (NEB) and further confirmed by vibrational frequency analysis.


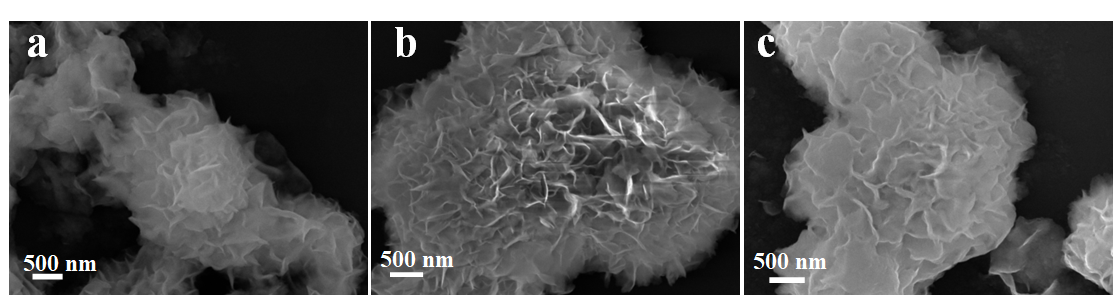


**Figure S1.** SEM images of (a) ZIS, (b) Vs-ZIS, (c) Vs-ZIS@Co-3.

**
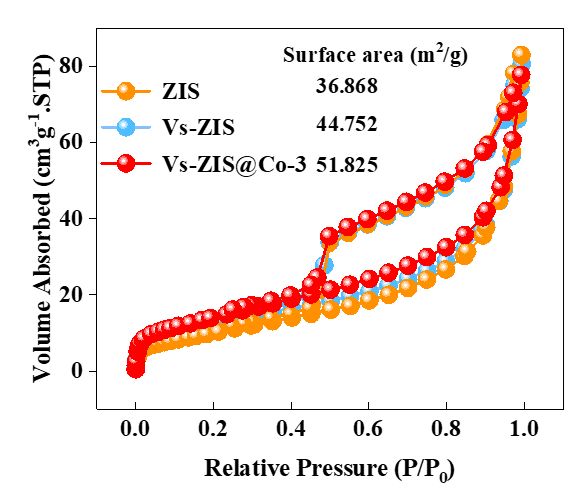
**

**Figure S2.** Nitrogen adsorption−desorption isotherms of ZIS, Vs-ZIS and Vs-ZIS@Co-3.


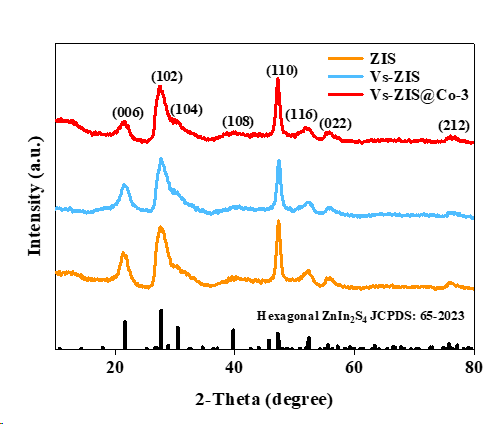


**Figure S3.** XRD spectra of ZIS, Vs-ZIS and Vs-ZIS@Co-3.

**Figure S4.** TEM images of (a) ZIS. (b) HRTEM image of ZIS. (c) Magnified view of the red box in (b).

**Figure S5.** TEM image of (a) Vs-ZIS. (b) HRTEM image of Vs-ZIS. (c) Magnified view of the yellow box in (b).

**Figure S6.** TEM image of (a) Vs-ZIS@Co-3. (b) HRTEM image of Vs-ZIS@Co-3. (c) Magnified view of the bule box in (b).

**Figure S7.** (a-e) EDS elemental mapping results of ZIS.

**Figure S8.** (a-e) EDS elemental mapping results of Vs-ZIS.

**Table S1.** EXAFS fitting parameters at the Co K-edge for various samples (Ѕ_0_^2^=0.85).

| **samples** | **shell** | **N^a^** | **R(Å)^b^** | **σ^2c^** | **ΔE_0_^d^** | **R factor** |
| --- | --- | --- | --- | --- | --- | --- |
| Co foil | Co-Co | 12 | 2.49±0.01 | 0.0065 | 7.8±0.3 | 0.0012 |
| CoS | Co-S | 6.3±0.3 | 2.21±0.01 | 0.0132 | -17.8±1.8 | 0.0132 |
|  | Co-Co | 1.5±0.8 | 2.75±0.04 | 0.0192 |  |  |
| Co_3_O_4_ | Co-O | 4.6±0.2 | 1.91±0.01 | 0.0029 | -8.4±0.6 | 0.0037 |
|  | Co-Co | 4.3±0.2 | 2.86±0.01 | 0.0045 |  |  |
|  | Co-Co1 | 7.7±0.4 | 3.36±0.01 | 0.0065 |  |  |
| Vs-ZIS@Co-3 | Co-S | 1.7±0.7 | 2.30±0.02 | 0.0096 | 0.3±1.6 | 0.0039 |
|  | Co-O | 4.4±0.6 | 2.08±0.02 | 0.0076 |  |  |

^a^N: coordination numbers; ^b^R: bond distance; ^c^σ^2^: Debye-Waller factors; ^d^ΔE_0_: the inner potential correction. R factor: goodness of fit.

**Figure S9.** EXAFS fitting curves in R-space of (a) Co foil, (b) Co_3_O_4_, (c) CoS, (d) Vs-ZIS@Co-3. EXAFS fitting curves in k-space of (e) Co foil, (f) Co_3_O_4_, (g) CoS, (h) Vs-ZIS@Co-3.

**Table S2.** Atomic concentrations (at.%) of Vs-ZIS@Co from XPS analysis.

| **Samples** | **Zn atomic concentration^&^** | **In atomic concentration^*^** | **Co atomic concentration^#^** |
| --- | --- | --- | --- |
| **Vs-ZIS@Co-1** | 9.98 | 30.57 | 0.34 |
| **Vs-ZIS@Co-2** | 9.49 | 30.67 | 0.72 |
| **Vs-ZIS@Co-3** | 9.65 | 29.79 | 0.99 |
| **Vs-ZIS@Co-4** | 9.39 | 29.91 | 1.57 |

^&^Zn atomic concentrations (at.%)$=\frac{\mathrm{Area}_{Zn}}{\mathrm{Area}_{Zn}+\mathrm{Area}_{In}+\mathrm{Area}_{S}+\mathrm{Area}_{Co}}$

^*^In atomic concentrations (at.%)$=\frac{\mathrm{Area}_{In}}{\mathrm{Area}_{Zn}+\mathrm{Area}_{In}+\mathrm{Area}_{S}+\mathrm{Area}_{Co}}$

^#^Co atomic concentrations (at.%)$=\frac{\mathrm{Area}_{Co}}{\mathrm{Area}_{Zn}+\mathrm{Area}_{In}+\mathrm{Area}_{S}+\mathrm{Area}_{Co}}$


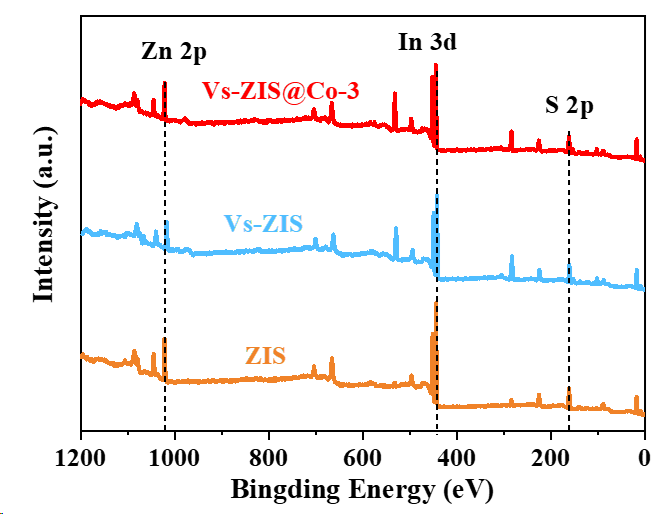


**Figure S10.** XPS spectras of full spectrum for ZIS, Vs-ZIS and Vs-ZIS@Co-3.

**Table S3.** XPS fitting data for peak area, the atomic ratios of S/In and Vs concentrations. The RSF (relative sensitivity factor) for Zn 2p, In 3d, S 2p and Co 2p are 28.72, 22.54, 1.677 and 19.16 respectively.

*S:In$=\frac{{Area}_{S}}{{Area}_{In}}$

Area: Area (RSF*T*MFP) of corresponding elements

^#^𝑉_𝑆_ 𝑐𝑜𝑛𝑐𝑒𝑛𝑡𝑟𝑎𝑡𝑖𝑜𝑛 (%) $=\frac{S:In\left( ZIS \right)-S:In\left( Modified ZIS \right)}{S:In\left( ZIS \right)}$

Modified ZIS: ZIS-3, ZIS-5 and Vs-ZIS@Co-y

| **Samples** | **Elements** | **Area/**  **(T*MFP)** | **Area/(RSF*T*MFP)** | **S/In^*^** | **Vs**  **concentration^#^** |
| --- | --- | --- | --- | --- | --- |
| **ZIS** | Zn 2p  In 3d  S 2P | 31497.6  76889.4  11654.7 | 1096.7  3411.2  6949.7 | 2.04 | 0 |
| **ZIS-3** | Zn 2p  In 3d  S 2p | 37679.4  102939.1  15341.3 | 1377.9  4566.9  9148.1 | 2.00 | 1.96% |
| **ZIS-5** | Zn 2p  In 3d  S 2p | 41012.4  120519.4  17284.5 | 1428.0  5346.9  10306.8 | 1.93 | 5.39% |
| **Vs-ZIS@Co-1** | Zn 2p  In 3d  S 2P  Co 2p | 29073.3  69085.1  9920.3  651.44 | 1012.3  3065.0  5915.5  34.0 | 1.93 | 5.39% |
| **Vs-ZIS@Co-2** | Zn 2p  In 3d  S 2P  Co 2p | 24030.2  68137.5  9936.4  1350 | 836.7  3023.0  5925.1  70.5 | 1.96 | 3.92% |
| **Vs-ZIS@Co-3** | Zn 2p  In 3d  S 2P  Co 2p | 27924.5  67665.1  10068.9  1916.0 | 972.3  3002.0  6004.1  100.0 | 2.00 | 1.96% |
| **Vs-ZIS@Co-4** | Zn 2p  In 3d  S 2P  Co 2p | 25922.7  72538.3  10847.2  3239.5 | 902.6  3218.2  6468.2  169.1 | 2.01 | 1.47% |


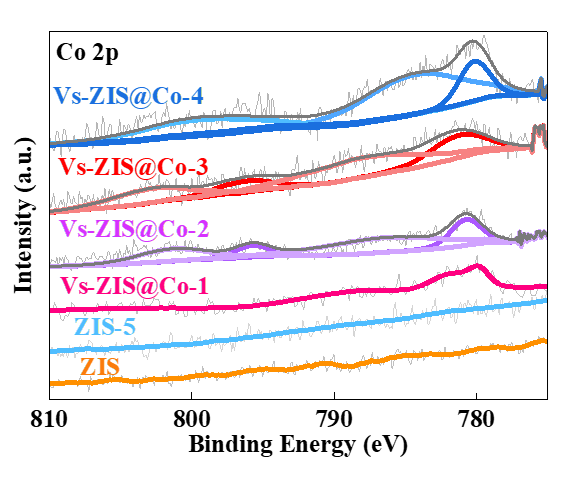


**Figure S11.** High-resolution XPS spectra of Co 2p peaks for ZIS, ZIS-5 and Vs-ZIS@Co-y (y = 1~4).

**Figure S12.** (a) Top view structural model and (b) corresponding charge density distributions for Vs-ZIS.


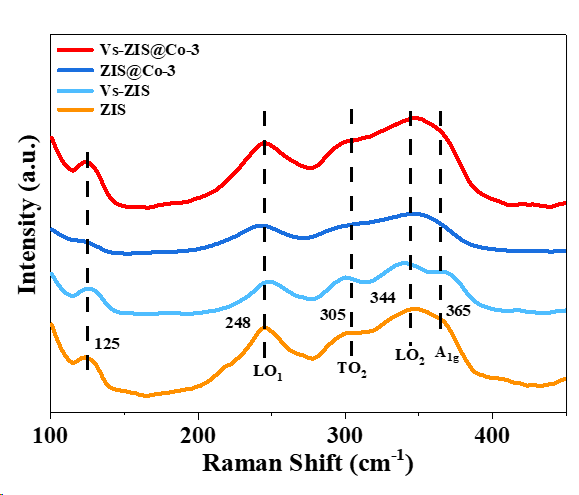


**Figure S13.** Raman spectra of ZIS, Vs-ZIS, ZIS@Co-3 and Vs-ZIS@Co-3.

**Figure S14.** (a) DRS spectra, (b) (*αhν*)^2^ versus (*hν*) plots, (c) Valence band spectra of X-ray photoelectron spectroscopy and (d) Band structures of ZIS, ZIS-3, ZIS-5, Vs-ZIS@Co-2 and Vs-ZIS@Co-3.

**Figure S15.** (a) PL spectra, (b) TRPL decay spectra of, (c) Photocurrent responses and (d) EIS Nyquist plots of ZIS, Vs-ZIS and Vs-ZIS@Co-3.

**Table S4.** The PL lifetime of ZIS, Vs-ZIS and Vs-ZIS@Co-3.

| **Sample**  **parameters** | **ZIS** | **Vs-ZIS** | **Vs-ZIS@Co-3** |
| --- | --- | --- | --- |
| 𝜏_1_ (ns) | 0.3351 | 0.2758 | 0.2613 |
| 𝜏_2_ (ns) | 1.6221 | 1.4416 | 1.3206 |
| 𝜏_3_ (ns) | 7.8108 | 6.2939 | 6.085 |
| A_1_ | 0.241 | 0.241 | 0.256 |
| A_2_ | 0.034 | 0.048 | 0.046 |
| A_3_ | 0.001 | 0.002 | 0.003 |
| 𝜏_ave_ (ns) ^#^ | 1.24 | 1.33 | 1.43 |

**^#^** The calculated formula:

$$\tau_{ave}=\frac{A_{1}\tau_{1}^{2}+A_{2}\tau_{2}^{2}+A_{3}\tau_{3}^{2}}{A_{1}\tau_{1}+A_{2}\tau_{2}+A_{3}\tau_{3}}$$

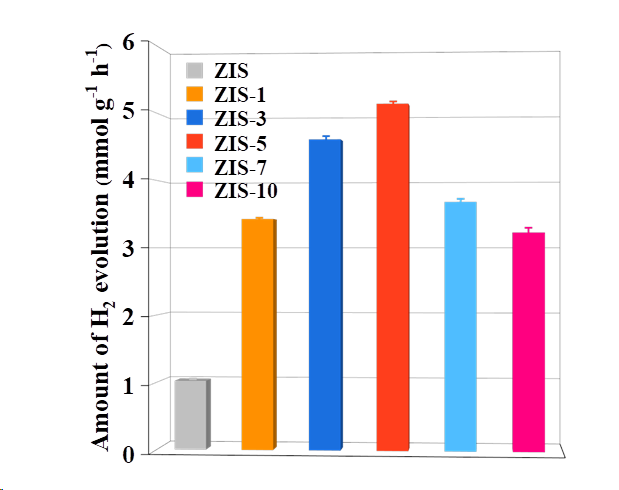


**Figure S16.** Amount of H_2_ evolution for ZIS and ZIS-x (x = 1, 3, 5, 7, 10) (Sacrificial agent: 0.5M ascorbic acid aqueous solution, 300 W Xenon lamp, λ > 420 nm)).


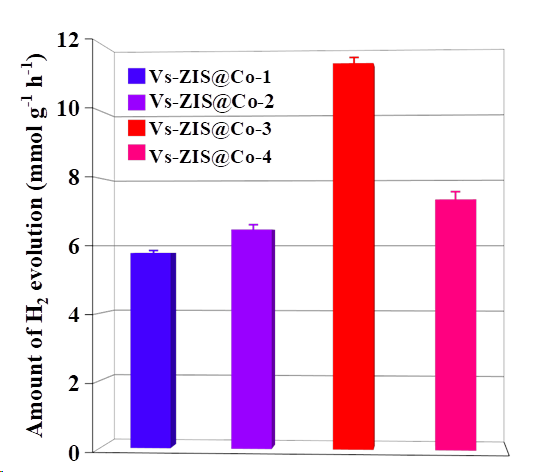


**Figure S17.** Amount of H_2_ evolution for Vs-ZIS@Co-y (y = 1~4) (Sacrificial agent: 0.5M ascorbic acid aqueous solution, 300 W Xenon lamp, λ > 420 nm).


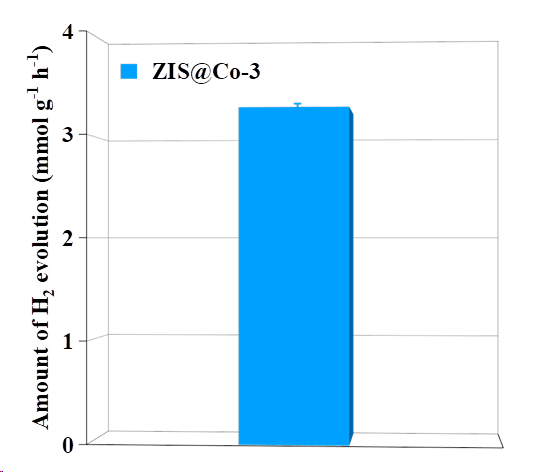


**Figure S18.** H_2_ evolution rate of ZIS@Co-3 (Sacrificial agent: 0.5M ascorbic acid aqueous solution, 300 W Xenon lamp, λ > 420 nm).

**Figure S19.** Mass spectra of (a) H2 (m/z=2) and (b) D2 (m/z=4) produced by Vs-ZIS@Co-3 photocatalysts during the water splitting reaction when H_2_O and D_2_O are used as proton sources, respectively.


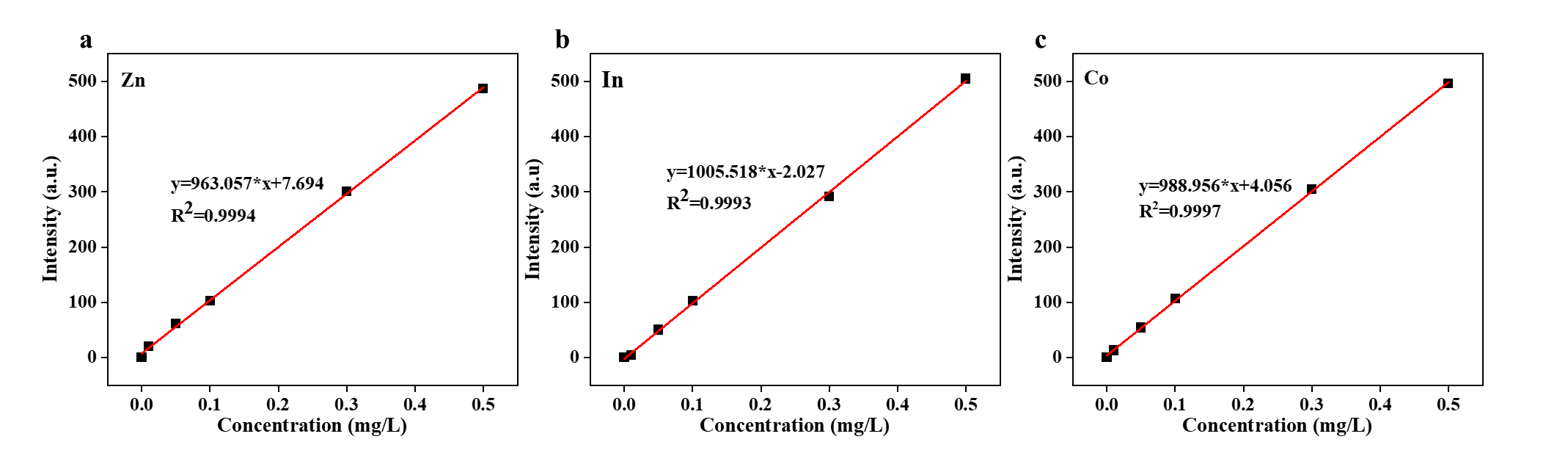


**Figure S****20.** Calibration curves (signal intensity *vs* concentration) of (a) Zn, (b) In and (c) Co in ICP-MS.

**Table S5.** The concentrations of metal ions and leaching rates for ZIS, Vs-ZIS and Vs-ZIS@Co-3 in each recycling reaction of photocatalytic hydrogen evolution from ICP-MS.

| **Parameter** | **Cycle** | **ZIS** | | **Vs-ZIS** | | **Vs-ZIS@Co-3** | | |
| --- | --- | --- | --- | --- | --- | --- | --- | --- |
|  |  | **Zn** | **In** | **Zn** | **In** | **Zn** | **In** | **Co** |
| **Concentrations (mg/L)** | 1st | 0.0474 | 0.0289 | 0.0477 | 0.0292 | 0.0463 | 0.0202 | 0.0052 |
|  | 2nd | 0.0528 | 0.0348 | 0.0541 | 0.0388 | 0.0523 | 0.0322 | 0.0055 |
|  | 3rd | 0.0699 | 0.0436 | 0.0716 | 0.0573 | 0.0672 | 0.0473 | 0.0071 |
|  | 4th | 0.0941 | 0.0662 | 0.0982 | 0.0709 | 0.0868 | 0.0607 | 0.0093 |
| **Leaching rate^#^ (%)** | 1st | 0.0948 | 0.0578 | 0.0954 | 0.0584 | 0.0926 | 0.0404 | 0.0104 |
|  | 2nd | 0.1056 | 0.0696 | 0.1082 | 0.0776 | 0.1046 | 0.0644 | 0.0110 |
|  | 3rd | 0.1398 | 0.0872 | 0.1432 | 0.1146 | 0.1344 | 0.0946 | 0.0142 |
|  | 4th | 0.1882 | 0.01324 | 0.1964 | 0.1418 | 0.1736 | 0.1214 | 0.0186 |

^#^$Leaching rate \left[ \% \right]=\frac{Concentration of metal ions}{50mglL\left( photocatalyst concentration \right)}\times100$


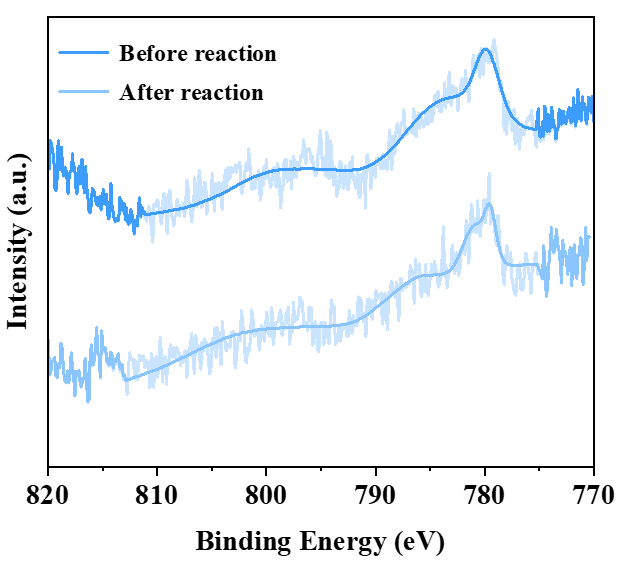


**Figure S2****1.** High-resolution XPS spectrum of Co 2p for the fresh and used Vs-ZIS@Co-3.

**Figure S22.** Performance comparison of photocatalytic water splitting in this work and some representative photocatalyst systems from 2018.

**Table S6.** Lists of some representative photocatalyst systems, reaction condition, rate of hydrogen and oxygen production, AQE, STH and corresponding reference from 2018.

| **Photocatalyst** | **Reaction environment (Light source, concentration of photocatalyst)** | **H_2_**  **(μmol g^-1^ h^-1^)** | **O_2_**  **(μmol g^-1^ h^-1^)** | **AQE**  **(%)** | **STH**  **(%)** | **Ref.** |
| --- | --- | --- | --- | --- | --- | --- |
| Pt-CoP/C_3_N_4_ | 300 W Xe arc lamp (λ> 400 nm),  30mg/60mL H_2_O | 26.25 | 12.50 | / | / | 1 |
| Pt/CdS/Al_2_O_3_ | 300 W Xe lamp (λ≥420 nm), 100mg/150mL H_2_O | 62.1 | Removed by  artificial gill | 0.22 (430 nm) | / | 2 |
| La_5_Ti_2_CuS_5_O_7_  /BiVO_4_ | Rh/Cr_2_O_3_, 300 W Xe lamp (λ＞420 nm), 100mg/100mL H_2_O | 22 | 11 | 4.9 (420 nm) | 0.11 | 3 |
| Cr_2_O_3_/Rh/IrO_2_-modified Y_2_Ti_2_O_5_S_2_ | 300 W Xe lamp (λ≥420 nm, H_2_O (pH 8.5) | 62 | 30 | 0.36 (420 nm)  0.23 (500 nm)  0.05 (600 nm) | 0.007 | 4 |
| g-C_3_N_4_/BiFeO_3_ | Pt/MnO_2_, 300 W Xe lamp (λ＞420 nm), 100mg/100mL H_2_O | 38.8 | 15.7 | 0.50 (420 nm) | / | 5 |
| PtS-ZnIn_2_S_4_/MnO_2_-WO_3_ | 300 W Xe arc lamp (λ≥ 400 nm),  30mg/60mL H_2_O | 14.8 | 5.6 | 0.50% (420 nm) | / | 6 |
| d-ZCS-P300 | 500 W Xe lamp (λ＞400 nm),30mg/120 mL H_2_O | 32.33 | 14.0 | 0.15 (420 nm) | / | 7 |
| MoS_2_-CdS/WO_3_-MnO_2_ | 300 W Xe lamp (λ＞420 nm),  100mg/100mL H_2_O | 0.5 | 0.26 | / | / | 8 |
| Ni_0.7_-ZIS | 300 W Xe arc lamp (λ> 400 nm),  30mg/60mL H2O | 84.3 | / | 19.7 (380 nm) | / | 9 |
| Pt-ZnIn_2_S_4_/RGO/  Co_3_O_4_-BiVO_4_(110) | Pt/Co_3_O_4_, 300 W Xe lamp (λ≥420 nm), 50mg/100mL H_2_O | 24.5 | 11.9 | / | / | 10 |
| Ag-ZnIn_2_S_4_ | 300 W Xe lamp (λ＞420 nm),  12mg/100mL H_2_O | 56.6 | 29.1 | 0.70 (405 nm)  0.57 (420 nm)  0.20 (450 nm) | 0.003 | 11 |
| d_Zni_-ZnIn_2_S_4_ | 300 W Xe lamp (λ＞420 nm), 50mg/120mL H_2_O | 42.8 | 19.1 | 1.51 (420 nm) | / | 12 |
| Al- ZnIn_2_S_4_ | 300 W Xe lamp (λ＞420 nm), 50mg/100mL H_2_O | 77.2 | 35.3 | 1.61 (420 nm) | / | 13 |
| 10%WP-Co/ZCS | 300 W Xe lamp (λ≥420 nm, light intensity 100mWcm^-2^), 20mg/100mL H_2_O | 61.0 | 32.5 | 27.6 (420 nm) | 1.57 | 14 |
| BiFeO_3_/ ZnIn_2_S_4_ | 300 W Xe lamp (λ＞420 nm), 12mg/100mL H_2_O | 87.3 | 42.3 | 1.12 (420 nm) | / | 15 |
| gZIS | 500 W Xe arc lamp (AM1.5), 30mg/60mL H_2_O | 36.04 | 18.96 | 0.17 (420 nm) | 0.002 | 16 |
| P-ZIS | 350 W Xe arc lamp (λ> 400 nm, 100 mW cm^−2^), 30mg/60mL H_2_O | 56 | / | 0.16 (420 nm) | / | 17 |
| Vs-ZIS@Co-3 | 300 W Xe lamp (λ＞420 nm),5mg/100mL H_2_O | 80.13 | 37.81 | 1.44 (380 nm)  1.31 (420 nm)  0.05 (500 nm) | 0.007 | This work |

**Text S1.** Apparent quantum eﬃciency (AQE)

The apparent quantum efficiency (AQE) for Vs-ZIS@Co-3 was measured under the exact same condition as photocatalytic hydrogen and oxygen evolution half-reaction as described, except that the solution consists of pure DI water without the presence of any sacriﬁcial reagent and the incident light was supplied by a 300W Xe lamp with different monochromatic light under various band pass ﬁlters (380, 420 and 500 nm) following the equation.

$$AQE=\frac{Number of reacted electrons}{Number of incident photos}\times100\%$$

$$=\frac{Number of evolved H_{2} molecules\times2}{Numbernof incident photons}\times100\%$$

Number of evolved H_2_ molecules = 2 × M × N_A_

Number of incident photons = Eλ/hc= Ptλ/hc

Where, M is the mole number of hydrogen molecules (mol), NA is Avogadro constant (6.022×10^23^ mol^-1^), h is Plank constant (6.626×10^-34^ J S), c is the speed of light (3×10^8^ m s^-1^), λ is the monochromatic light wavelength (nm), P is the average intensity of irradiation, S is the irradiation area (cm^2^), and t is the photoreaction time (s).

**Text S2.** Solar-to-hydrogen conversion efficiency (STH)

The conversion efficiency of STH was determined using an AM1.5 filter under 1 solar irradiation with the following equation:


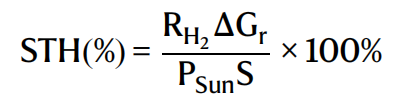


whereby R_H2_ = rate of H_2_ evolution (in mol∙s^−1^), ΔGr= Gibbs free energy change of water splitting reaction and P_sun_ = energy flux of the incident ray.

**Table S7.** AQE at different wavelengths for Vs-ZIS@Co-3 in water splitting.

| **Wavelength (nm)** | **H_2_ Evolved (μmol)** | **Light Intensity (mW)** | **AQE** |
| --- | --- | --- | --- |
| λ=380 | 5.31 | 32.17 | 1.44% |
| λ=420 | 5.23 | 36.50 | 1.31% |
| λ=500 | 0.27 | 39.50 | 0.05% |

**Figure S23.** Static contact angle of (a) ZIS, (b) Vs-ZIS, (c) ZIS@Co-3 and (d) Vs-ZIS@Co-3.


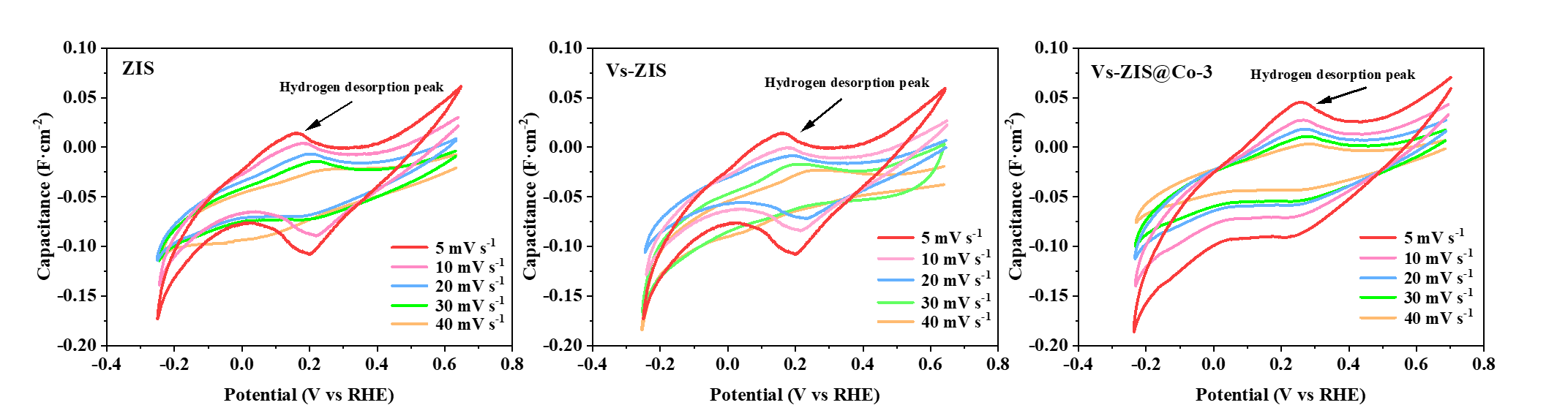


**Figure S24.** Cyclic voltammetry of (a) ZIS, (b) Vs-ZIS, and (c) Vs-ZIS@Co-3 in Ar-saturated 0.5M H_2_SO_4_ with a scan rate of 5 ~ 40 mVs^-1^.

**Table S8.** Optimized structural model of ZIS, Vs-ZIS and Vs-ZIS@Co-3.

| **Sample** | **Side view** | **Top view** |
| --- | --- | --- |
|  | **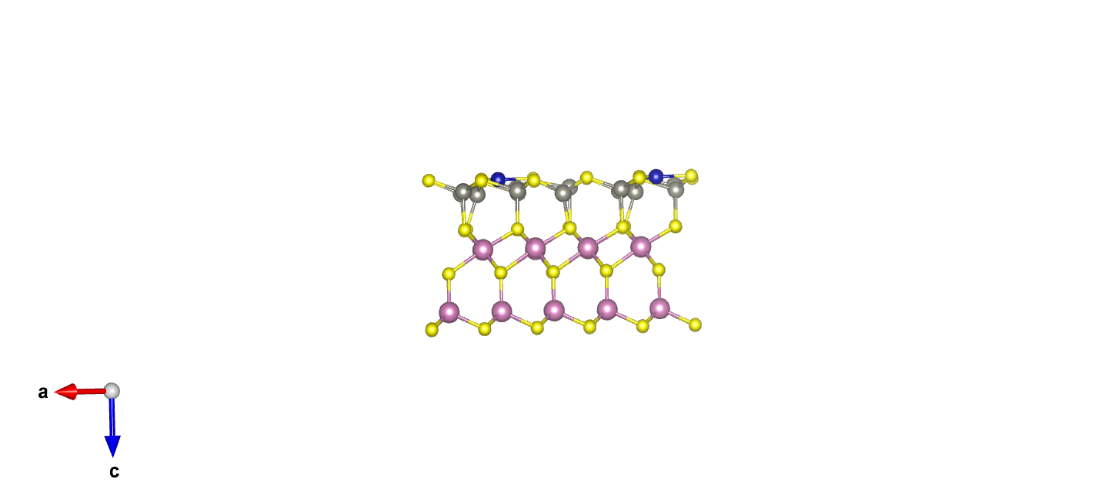** | 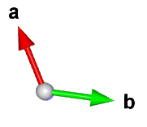 |
| **ZIS** | 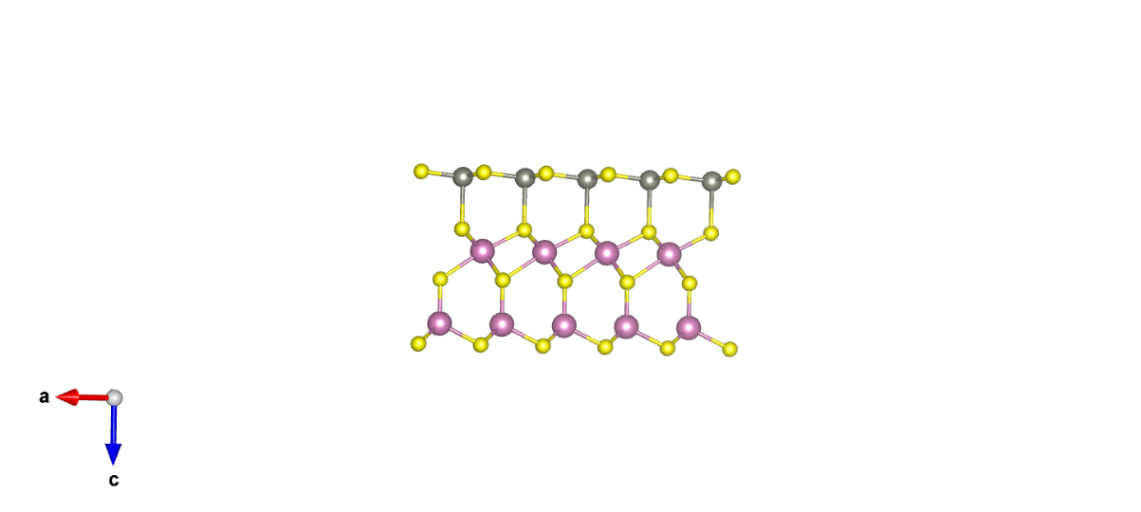 | 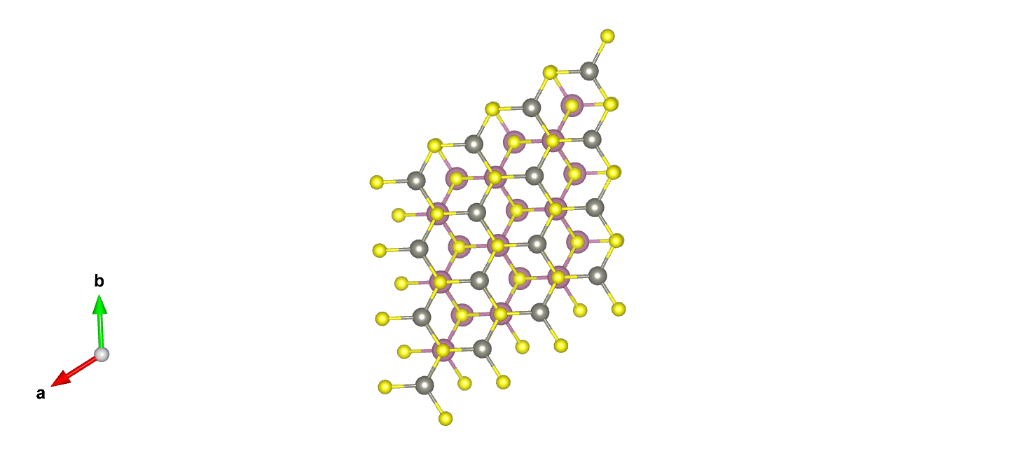 |
| **Vs-ZIS** | 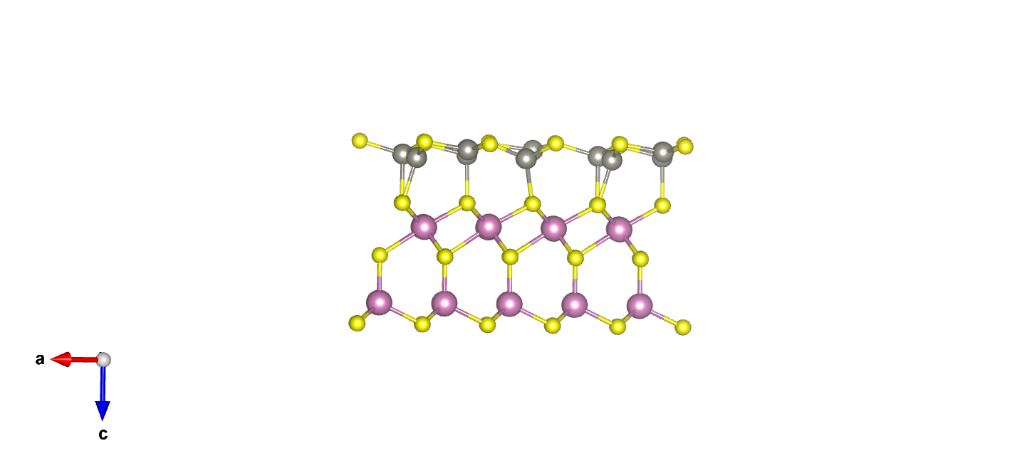 | 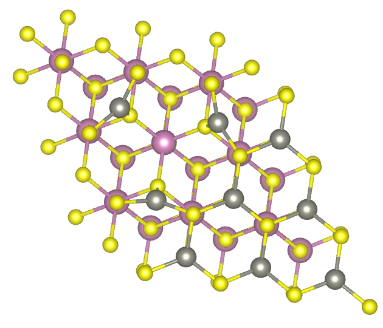 |
| **Vs-ZIS@Co-3** | 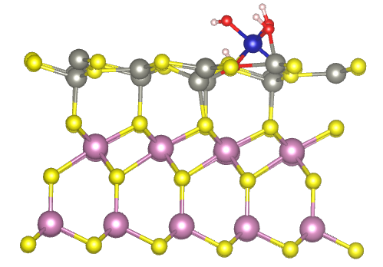 | 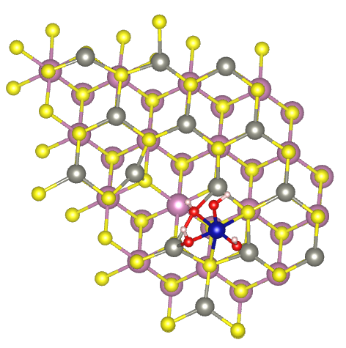 |

**Table S9.** Optimized structural model of adsorbed H_2_O, H* and HO* on ZIS, Vs-ZIS and Vs-ZIS@Co-3.

| **Adsorbed**  **species**  **Sample** | ***H_2_O** | **H*+HO*** | |
| --- | --- | --- | --- |
| **ZIS** | 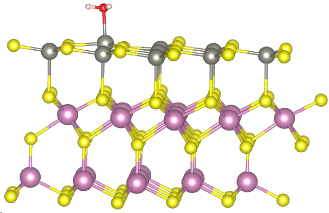 | | 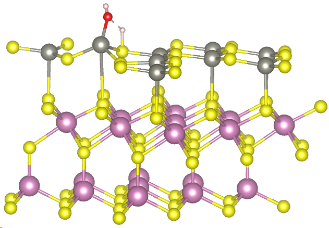 |
| **Vs-ZIS** | 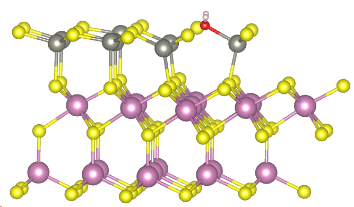 | | 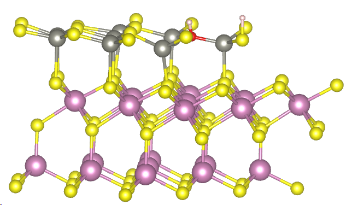 |
| **Vs-ZIS@Co-3** | 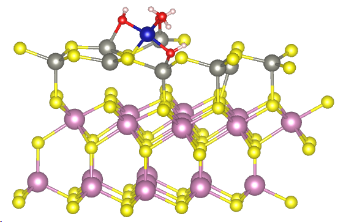 | | 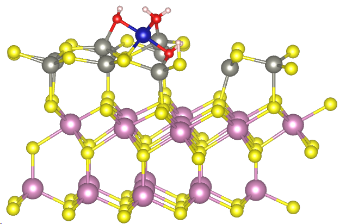 |


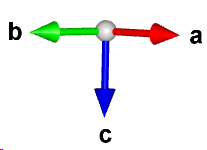


**Table S10.** Optimized structural model of adsorbed HO*, O* and HOO* onto ZIS, Vs-ZIS and Vs-ZIS@Co-3.

| **Adsorbed species**  **Sample** | **HO*** | | **O*** | **HOO*** |
| --- | --- | --- | --- | --- |
| **ZIS 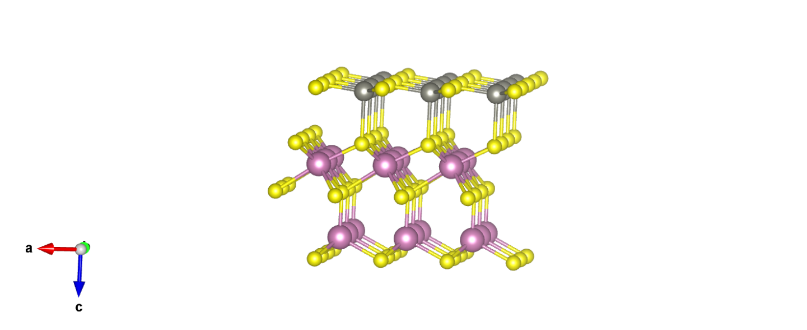** | | 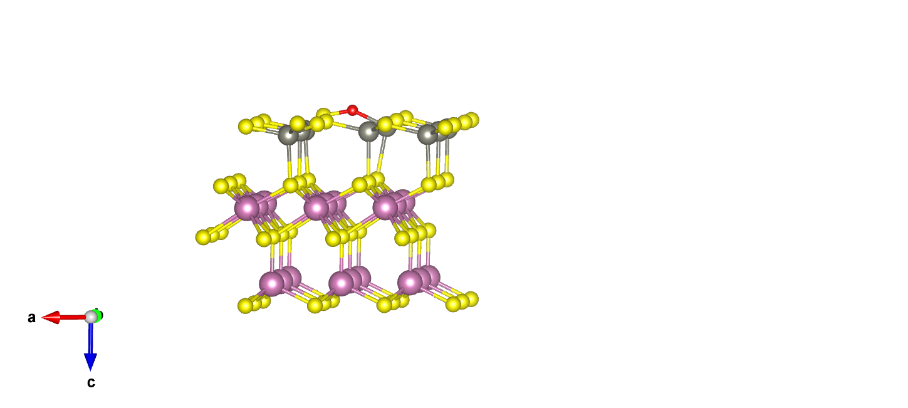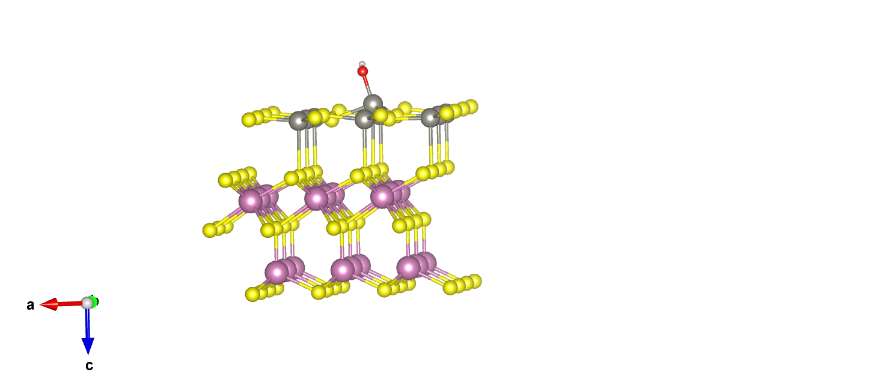 | 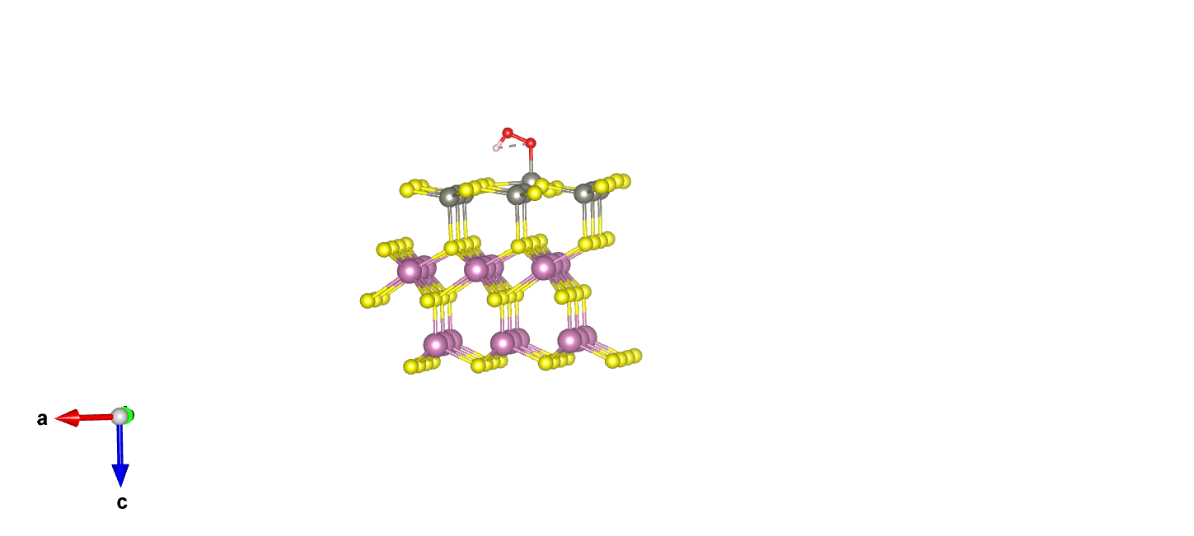 |  |
| **Vs-ZIS 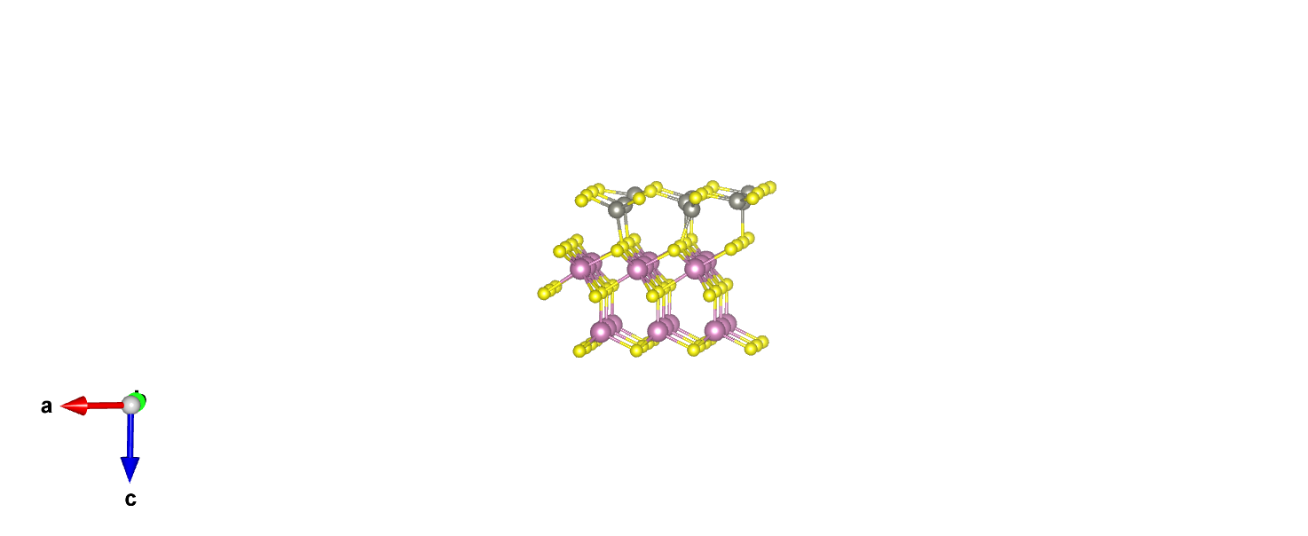** | | 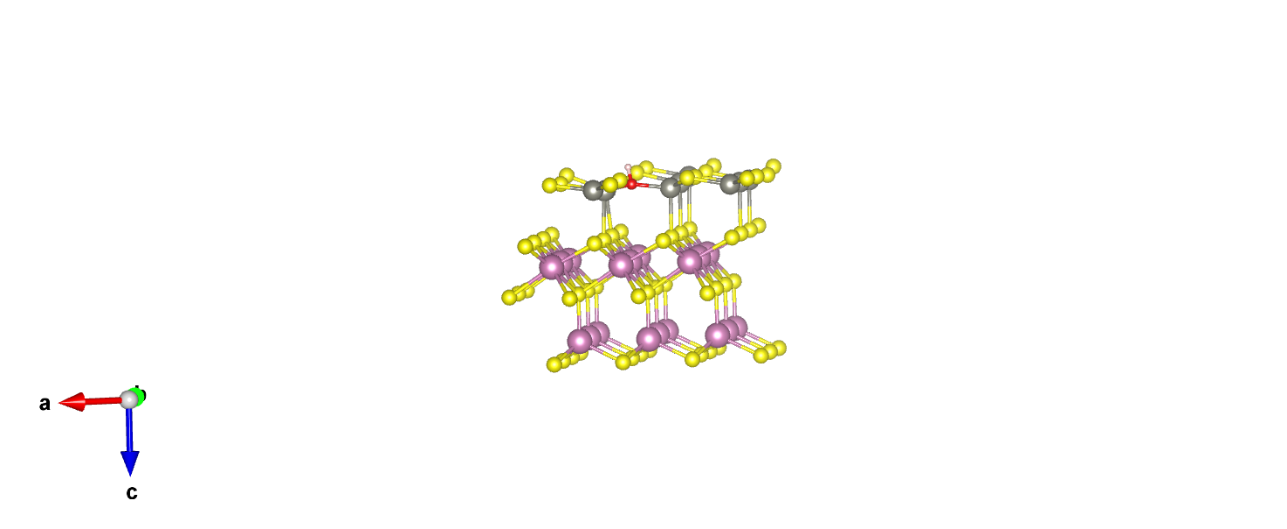 | 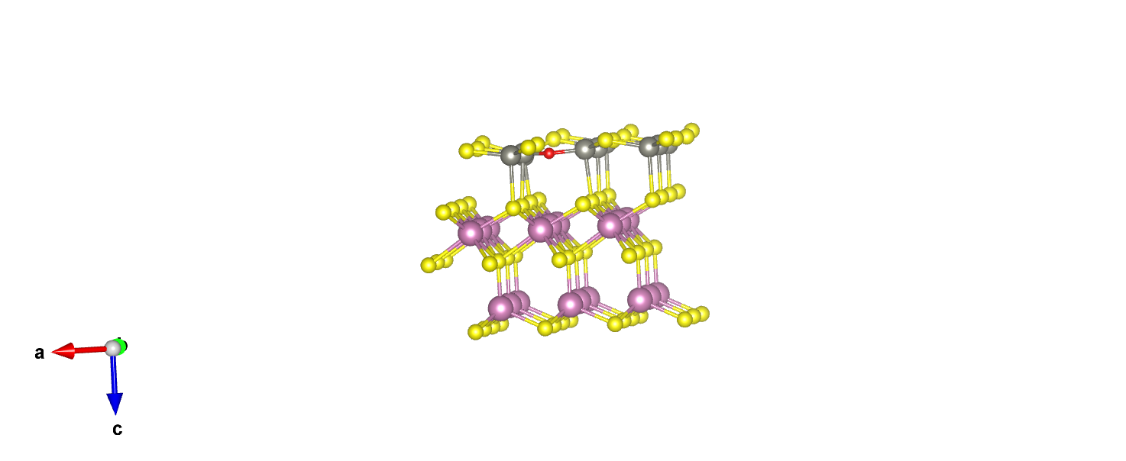 | 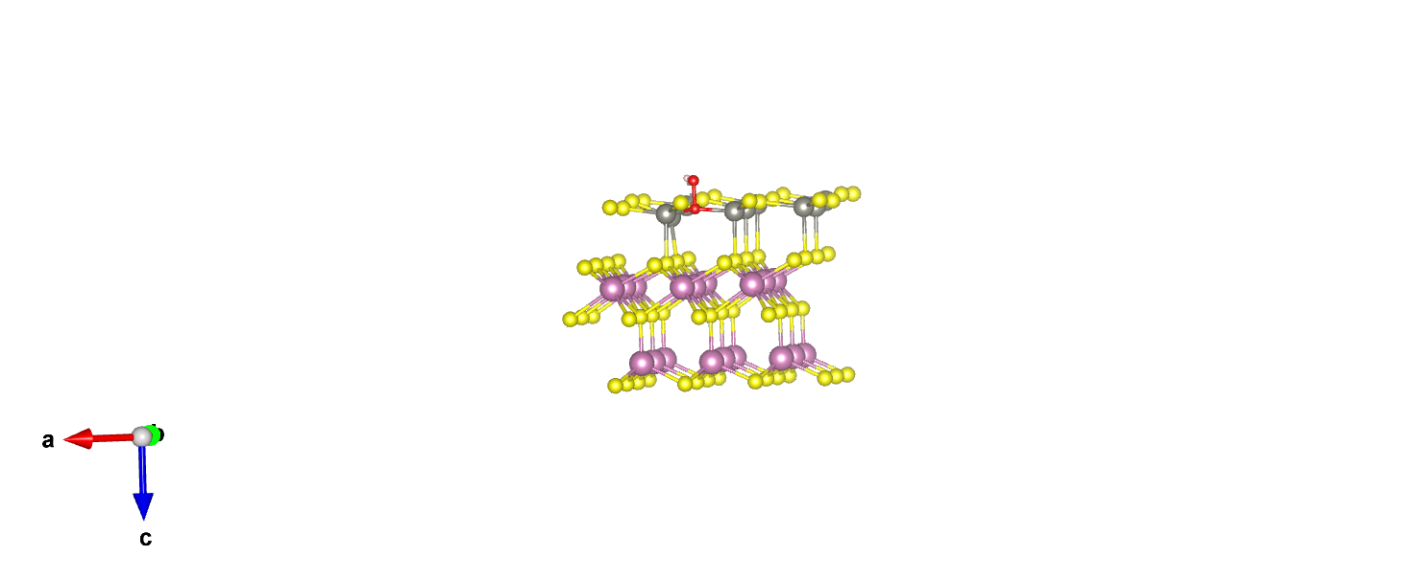 |
| **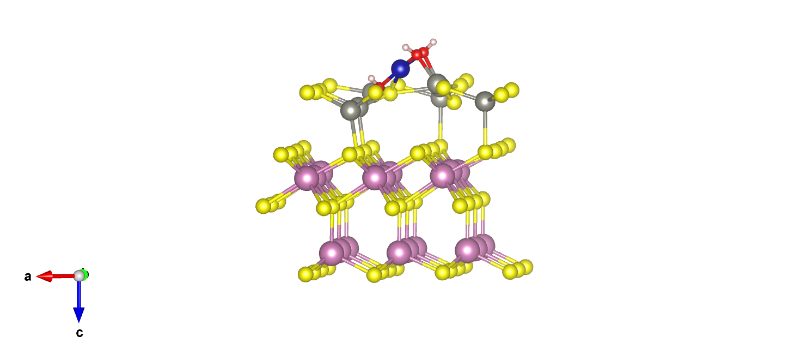Vs-ZIS@Co-3** | | 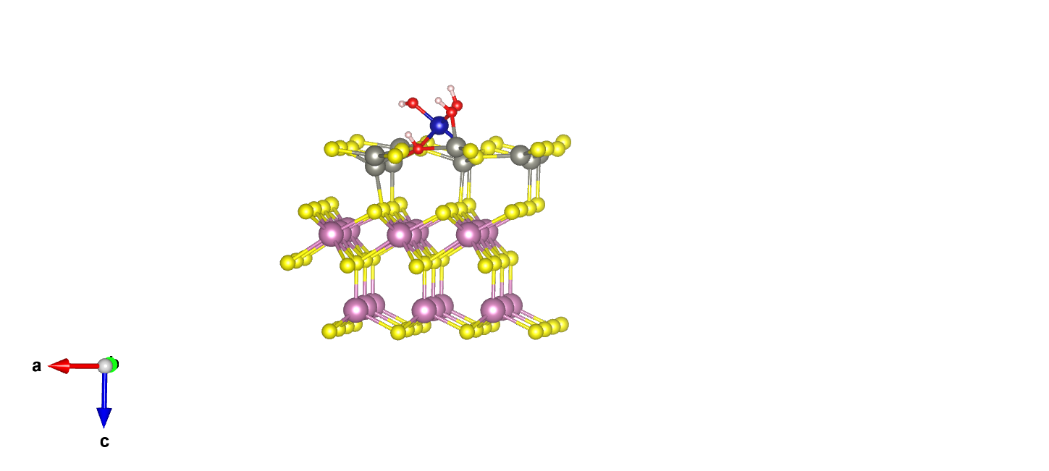 | 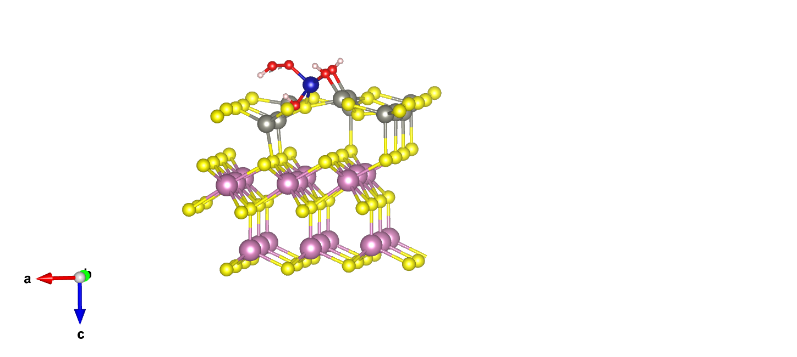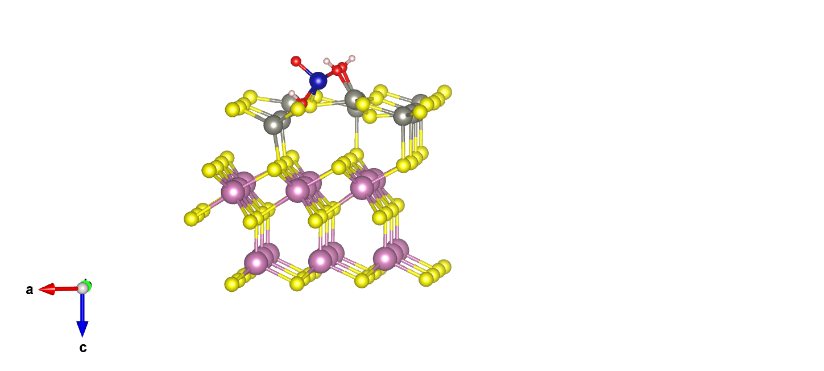 |  |


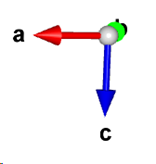


The OER process can be evaluated on the basis of four basic steps in the adsorption of HO*, O* and HOO* intermediates to an active surface. Therefore, the respective Gibbs free energy of each step can be calculated with reference to the standard Gibbs free energy of water splitting, summarized as:

∆G_OER1_ = E_HO*_ +$\frac{1}{2}$E_H2_ − E_H2O_ − E^∗^ + (∆ZPE − T∆S)_OER1_ – eU

∆G_OER2_ = E_O*_ +$\frac{1}{2}$E_H2_ − E_HO*_ + (∆ZPE − T∆S)_OER2_ – eU

∆G_OER3_ = E_HOO*_ +$\frac{1}{2}$E_H2_ − E_O*_ − E_H2O_ + (∆ZPE − T∆S)_OER3_ – eU

∆G_OER4_ = (4.92 + 2E_H2O_ −$\frac{3}{2}$E_H2_) + E^∗^ − E_HOO*_ + (∆ZPE − T∆S)_OER4_ – eU

in which U = 0, ∆G_2H2O→O2+2H2_ = 4.92 eV^2^, and E_HO*_, E_O*_, and E_HOO*_ determine the energies of adsorption on the HO*, O*, and HOO* surfaces, respectively. The OER overpotential (η_OER_) is defined as:

η_OER_= {max(|∆G_OER1_|,|∆G_OER2_|,|∆G_OER3_|,|∆G_OER4_|)}/e

**Reference**

1. Zhiming Pan, Yun Zheng, Fangsong Guo, Pingping Niu, Prof. Xinchen Wang. *ChemSusChem* **2017**, 10, 87-90.
2. Xiaofeng Ning, Wenlong Zhen, Yuqi Wu, Gongxuan Lu. *Appl. Catal. B: Environ.* **2018**, 226, 373-383.
3. Song Sun, Takashi Hisatomi, Qian Wang, Shanshan Chen, Guijun Ma, Jingyuan Liu, Swarnava Nandy, Tsutomu Minegishi, Masao Katayama, and Kazunari Domen. *ACS Catal.* **2018**, 8, 1690-1696.
4. Qian Wang, Mamiko Nakabayashi, Takashi Hisatomi, Song Sun, Seiji Akiyama, Zheng Wang, Zhenhua Pan, Xiong Xiao, Tomoaki Watanabe, Taro Yamada, Naoya Shibata, Tsuyoshi Takata & Kazunari Domen. *Nat. mater.* **2019**, 18, 827-832.
5. Hadis Sepahvand, Shahram Sharifnia. *Int. J. Hydrogen Energy* **2019**, 44, 23658-23668.
6. Yao Ding, Dingqiong Wei, Rong He, Rusheng Yuan, Tengfeng Xie, Zhaohui Li. *Appl. Catal. B: Environ.* **2019**, 258, 117948.
7. Boon-Junn Ng, Lutfi Kurnianditia Putri, Xin Ying Kong, Pooria Pasbakhsh, Siang-Piao Chai. *Appl. Catal. B: Environ.* **2020**, 262, 118309.
8. Dingqiong Wei, Yao Ding, Zhaohui Li. *Int. J. Hydrog. Energy* **2020**, 45, 17320-17328.
9. Xiaowei Shi, Liang Mao, Chao Dai, Ping Yang, Junying Zhang, Fuyuan Dong, Lingxia Zheng, Mamoru Fujitsuka, Huajun Zheng. *J. Mater. Chem. A* **2020**, 8, 13376-13384.
10. Man Ou, Jinke Li, Mei Geng, Jing Wang, Shipeng Wan, Qin Zhong. *Catal. Lett.* **2021**, 151, 2570-2582.
11. Rongrong Pan, Min Hu, Jia Liu, Dongfeng Li, Xiaodong Wan, Hongzhi Wang, Yuemei Li, Xiuming Zhang, Xiuli Wang, Jun Jiang, Jiatao Zhang. *Nano Lett.* **2021**, 21, 6228-6236.
12. Bojing Sun, Jiaqi Bu, Xiaoyu Chen, Dingge Fan, Siwei Li, Zhenzi Li, Wei Zhou, Yunchen Du. *Chem. Eng. J.* **2022**, 435, 135074.
13. Bojing Suna, Dingge Fana, Xiaoyu Chena, Zhenzi Lib, Wei Zhou, Yunchen Du. *Mater. Chem. Front.* **2022**, 6, 1795-1802.
14. Xuqiang Hao, Yifan Shao, Dingzhou Xiang, Zhiliang Jin. *Sol. Energy Mater. Sol. Cells* **2022**, 248, 111970.
15. Jian Zhang, Ying Zhang, Lutao Li, Wei Yan, Haiyun Wang, Weiwei Mao, Yan Cui, Yonghua Li, Xinbao Zhu. *J. Mater.Chem. A* **2023**, 11, 434-446.
16. Wei-Kean Chong, Boon-Junn Ng, Yong Jieh Lee, Lling-Lling Tan, Lutfi Kurnianditia Putri, Jingxiang Low, Abdul Rahman Mohamed, Siang-Piao Chai. *Nat. Commun.* **2023**, 14, 7676.
17. Boon-Junn Ng, Wei-Kean Chong, Lutfi Kurnianditia Putri, Xin Ying Kong, Jingxiang Low, Hing Wah Lee, Lling-Lling Tan, Wei Sea Chang, Siang-Piao Chai. *J. Mater. Chem. A* **2023**, 11, 17079-17090.
